# Identification of a Novel Radiosensitivity‐Related Signature and Validation of GPX8 in Regulating the Radiosensitivity of Glioma

**DOI:** 10.1002/cns.70900

**Published:** 2026-04-28

**Authors:** Gan Tao, Xiuli Guo, Fajian He, Sisi Yan, Qingwei Wang, Xiaowan Guo, Kehua Hu, Conghua Xie, Qiuji Wu, Yahua Zhong

**Affiliations:** ^1^ Department of Radiation and Medical Oncology, Hubei Key Laboratory of Tumor Biological Behavior, Hubei Provincial Clinical Research Center for Cancer Zhongnan Hospital of Wuhan University Wuhan China

**Keywords:** apoptosis, DNA damage, glioma, GPX8, radiosensitivity

## Abstract

**Background:**

Radioresistance is fundamental to glioma progression and poor prognosis. Understanding its underlying mechanisms and identifying novel therapeutic targets through elucidating key molecules in glioma radiosensitivity pathways are therefore of significant clinical importance.

**Methods:**

Radiosensitivity‐related genes were identified based on radiotherapy response, glioma stemness, and prognosis. A predictive signature was constructed using Lasso‐Cox regression and validated via clinicopathological, functional enrichment, immune infiltration, and correlation analyses. GPX8 expression and prognostic significance were assessed by tissue microarray. In vitro functional and radiobiological assays, complemented by in vivo subcutaneous xenograft models using BALB/c nude mice (treated with or without radiotherapy), evaluated the role of GPX8 in regulating malignant progression and radiosensitivity in glioma.

**Results:**

The radiosensitivity‐related signature demonstrated significant potential in predicting glioma malignancy and prognosis, serving as an indicator of the mesenchymal subtype and contributing to the maintenance of an immunosuppressive microenvironment. GPX8 was overexpressed in high‐grade gliomas and correlated with recurrence and poor survival. Knockdown of GPX8 suppressed the malignant biological behaviors of glioma cells. Radiation upregulated GPX8 expression while GPX8 knockdown significantly enhanced the cytotoxicity of radiation and induced apoptosis by promoting oxidative stress and DNA damage. Suppression of GPX8 effectively potentiated radiosensitivity in murine xenograft models and reduced intratumoral infiltration of tumor‐associated macrophages.

**Conclusions:**

The radiosensitivity‐related signature serves as a significant predictor for assessing glioma malignancy and prognosis. GPX8 acts as a key regulator of malignant phenotypes and radiosensitivity in glioma, positioning it as a promising therapeutic target to counteract both malignant progression and radioresistance.

## Graphical abstract

The technical roadmap of this study involves the identification of a novel radiosensitivity‐related signature and the validation of GPX8 in regulating the radiosensitivity of glioma.

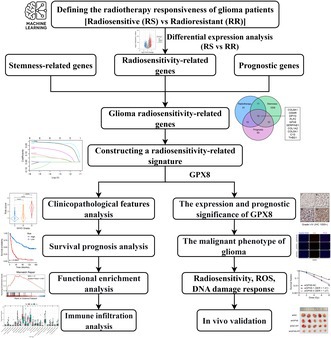

**Abbreviations**
1p/19q: Chromosomal arms 1p and 19q
ATCC: American Type Culture Collection
AUC: Area under curve
BP: Biological processes
CCK‐8: Cell counting kit‐8 assay
CDF: Cumulative distribution function
CGCs: Conventional glioma cells
CGGA: Chinese Glioma Genome Atlas
CL: Classical
CNS: Central nervous system
CSCs: Cancer stem cells
DFS: Disease‐free survival
EMT: Epithelial‐mesenchymal transformation
ER: Endoplasmic reticulum
GBM: Glioblastoma multiforme
GEO: Gene Expression Omnibus
GPX8: Glutathione Peroxidase 8
GSCs: Glioma stem cells
GSEA: Gene Set Enrichment Analysis
GSVA: Gene Set Variation Analysis
HGG: High‐grade glioma
IDH1/2: Isocitrate dehydrogenase 1 and 2
LGG: Low‐grade glioma
MES: Mesenchymal
MGMT: O‐6‐methylguanine‐DNA methyltransferase
NE: Neural
OS: Overall survival
PCA: Principal component analysis
PDI: Protein disulfide isomerase
PI: Pyridine iodide
PMT: Proneural‐mesenchymal transformation
PN: Proneural
ROS: Reactive oxygen species
RR: Radioresistant
RS: Radiosensitive
RT: Radiotherapy
SER: Sensitization enhancement ratio
SF: Survival fraction
si‐RNA: small interfering RNA
TAM: Tumor‐associated macrophage
TCGA: The Cancer Genome Atlas
TMZ: Temozolomide
Tregs: Regulatory T cells
WHO: World Health Organization

## 1 Introduction

Glioma is the most common primary malignant tumor of the central nervous system (CNS), with high recurrence and mortality rates [1]. According to the 2021 CNS tumors classification by the World Health Organization (WHO), gliomas are classified into Grades I‐IV [2]. As the most malignant subtype of glioma, glioblastoma multiforme (GBM, Grade IV) typically exhibits high proliferative activity and invasive ability. The current standardized treatment includes maximum safe surgical resection of the tumor lesion, followed by adjuvant therapy with temozolomide (TMZ) and/or radiotherapy (RT). Despite great efforts, tumor recurrence and chemoradiotherapy resistance still result in poor prognosis for patients, with a median survival period generally not exceeding 2 years [3, 4].

Radiotherapy is a cornerstone treatment for glioma, inducing tumor cell death through oxidative stress and DNA damage. However, nearly all high‐grade gliomas recur locally within months, due in significant part to their ability to develop radioresistance via complex molecular mechanisms [5]. For instance, a subpopulation within tumor tissue possesses multi‐lineage differentiation potential and self‐renewal capacity: cancer stem cells (CSCs). These cells exhibit more efficient DNA damage repair and enhanced anti‐apoptotic properties compared to conventional tumor cells, enabling them to preferentially survive radiotherapy and other treatments [6]. Key molecules regulating glioma stemness, such as EZH2, CD44, and YY1, have been identified and shown to play critical roles in modulating glioma radiosensitivity [7, 8, 9].

Differences in molecular characteristics such as isocitrate dehydrogenase 1 and 2 (IDH1/2) mutation status, chromosomal arms 1p and 19q (1p/19q) co‐deletion status, and O‐6‐methylguanine‐DNA methyltransferase (MGMT) promoter methylation status also affect the responsiveness of glioma to chemoradiotherapy and the prognosis of patients [10]. In addition, Verhaak et al. classified the genomic abnormalities of GBM into four subtypes based on The Cancer Genome Atlas (TCGA) network: mesenchymal (MES), classical (CL), neural (NE), and proneural (PN) subtypes [11]. Numerous studies have demonstrated that gliomas of the MES subtype exhibit greater malignancy and comparative radioresistance than those of the PN subtype. The stemness of tumor cells and the malignant biological behaviors of glioma stem cells (GSCs) are also vastly different between the two subtypes [12, 13].

GPX8 is the newly discovered member of the glutathione peroxidase (GPX) family, most members of which regulate intracellular oxidative stress processes by utilizing glutathione as a reducing agent to eliminate cellular peroxides. Among them, GPX4 is a well‐established key regulator suppressing ferroptosis [14]. Recent studies have revealed high expression of GPX8 in various tumor tissues, suggesting its potential significance, including promoting tumor invasion and metastasis by regulating epithelial‐mesenchymal transformation (EMT) in lung cancer and breast cancer [15, 16], promoting malignant progression by regulating apoptosis and autophagy in esophageal cancer [17], and participating in the regulation of lipid metabolism in renal clear cell carcinoma [18]. In glioma, Chen et al. identified GPX8 as a pan‐apoptotic related molecule that could promote the migration of microglia and mediate immunotherapy responses [19]. However, there have been no relevant reports on the relationship between GPX8 and tumor radiosensitivity, especially in the study of glioma radioresistance.

In this study, we employed bioinformatics analysis of transcriptomic data to identify key radiosensitivity‐related genes based on patient responsiveness to radiotherapy, glioma stemness, and prognostic characteristics. Subsequently, we constructed a radiosensitivity‐related gene signature and validated its association with clinicopathological features and prognostic value. GPX8 was then selected as the core molecule for further experimental investigation. We demonstrated that downregulating GPX8 expression significantly inhibited malignant phenotypes in glioma cells and reversed mesenchymal transition. Crucially, GPX8 knockdown enhanced the cytotoxic effects of radiation in glioma cells by promoting oxidative stress and DNA damage. Moreover, suppression of GPX8 effectively potentiated radiosensitivity and reduced intratumoral infiltration of tumor‐associated macrophages in murine xenograft models. Collectively, our findings identify GPX8 as a potential therapeutic target for combining with radiotherapy in glioma.

## 2 Methods

### 2.1 Data Source

The RNA‐seq expression profiles and clinical information of CGGA325 and CGGA693 datasets were obtained from the Chinese Glioma Genome Atlas (CGGA) [20] database. The RNA‐seq expression profiles and clinical information of 678 glioma patients were obtained from the TCGA database. The GSE23806 dataset (including microarray data of 27 glioma stem cells and 32 conventional glioma cell lines) was downloaded from the Gene Expression Omnibus (GEO) database.

### 2.2 Bioinformatics Analysis

R 4.4.1 was used to organize and analyze the data, and the RNA‐seq expression profile was standardized into log2 (TPM + 1) format. We utilized the R package “ConsensusClusterPlus” to perform unsupervised consensus clustering in the CGGA dataset. In differential expression analysis, the “DESeq2” package was applied to the raw count value, and the “limma” package was applied to microarray data. The “survival” package was used in Lasso‐Cox regression, Kaplan–Meier survival analysis, univariate and multivariate Cox regression, and the “survival ROC” package was used in fitting the survival ROC curve. When conducting survival analysis, we excluded patients with an overall survival (OS) of less than 30 days. When constructing the radiosensitivity‐related signature, we calculated the Risk‐score using the following formula: Risk‐score = $\sum_{i}^{n}\mathrm{Xi}*\mathrm{Yi}$ (X and Y respectively represent the λ coefficient in Lasso‐Cox regression and expression of radiosensitivity‐related genes). Gene Set Enrichment Analysis (GSEA) and Gene Set Variation Analysis (GSVA) respectively used the “clusterProfiler” and “GSVA” packages. The comparative analysis of GPX8 expression between normal brain tissue and glioma was performed using the online database GEPIA.

### 2.3 Glioma Tissue Microarray and Immunohistochemical (IHC) Staining

The tissue microarray containing tumor samples from 177 glioma patients was obtained from Xinchao Biotechnology (Shanghai, China). All patients have signed informed consent forms and were approved by the company's ethics committee. Immunohistochemical staining was performed as follows: Tissue sections underwent baking, dewaxing, and antigen retrieval. Slides were incubated overnight at 4°C with primary antibody against GPX8, followed by a 45 min incubation with secondary antibody at room temperature. Staining was completed using a DAKO fully automated immunohistochemistry system for blocking, secondary antibody application, and DAB development. Sections were counterstained with hematoxylin and mounted. The interpretation of the results was scored by two or more experienced professional pathologists on the staining intensity and positivity rate. The final score was calculated as the product of these two parameters. After excluding samples with tissue detachment, GPX8 expression data were successfully obtained from 158 patients. The list of antibodies used in this study is provided in (Table S2).

### 2.4 Cell Culture

The U87, U251, LN229, and SHG‐44 glioma cell lines were derived from the Cell Bank of the Committee on Type Culture Collection, Chinese Academy of Sciences, and cultured in DMEM high glucose medium containing 10% fetal bovine serum (FBS) and 1% penicillin–streptomycin at 37°C, 5% CO2 humidified incubator.

### 2.5 Cell Transfection

The small interfering RNA (siRNA) knockdown of GPX8 was synthesized by Aoke Biotechnology (Wuhan, China). The specific sequence is shown in Table S1. According to the manufacturer's instructions, we used jetPRIME siRNA transfection reagent (Polyplus, France) to transfect U251 and LN229 cells and collected total RNA and protein for detection 48‐72 h after transfection.

### 2.6 Reverse Transcription Quantitative Polymerase Chain Reaction (RT‐qPCR)

Total RNA was extracted from cells using the Trizol method, and cDNA of sufficient concentration was obtained by reverse transcription according to the manufacturer's instructions (ABclonal Biotechnology, Wuhan, China). After combining cDNA, SYBR, and relevant primers into a suitable reaction system, subsequent amplification was performed using a standardized qPCR program. The relative expression of genes was calculated using the 2^−ΔΔCT^ method (normalized with β‐actin expression as a reference). The primer sequence used is shown in Table S1.

### 2.7 Western Blotting

The total protein of cells was extracted with RIPA lysate, and the protein was separated on 10% or 12.5% PAGE gel and transferred to the PVDF membrane. After being sealed with 5% skimmed milk for 2 h, it was incubated with the primary antibody at 4°C overnight. After cleaning, the secondary antibody was incubated at room temperature for 1 h. The protein bands were visualized by chemiluminescence analysis on a developing instrument. The list of antibodies used in this study is provided in (Table S2).

### 2.8 Cell Counting Kit‐8 (CCK‐8) Assay

Cells transfected for 48 h were collected and seeded into a 96‐well plate, with 3000 cells per well and 5 replicates per group. Then we added 10% CCK‐8 reagent to each well 24, 48, and 72 h after inoculation, incubated at 37°C for 1 h, and measured the absorbance (OD, 450 nm) of each well using an enzyme‐linked immunosorbent assay (ELISA) reader.

### 2.9 EdU Cell Proliferation Assay

The EdU Cell Proliferation Imaging Analysis Kit was purchased from Abbkine Biotechnology (Wuhan, China). Glioma cells transfected for 48 h were seeded into a 96‐well plate, with 5000 cells per well and 3 replicates per group. After 24 h of cultivation in a 37°C incubator, the cells were sequentially labeled, fixed, stained, and counterstained with EdU, following the manufacturer's instructions for use. Finally, a conventional fluorescence microscope was used for cell photography.

### 2.10 Transwell Assay

We performed cell invasion and migration assays using Transwell chambers in a 24‐well plate. 48 h after transfection, the cells were resuspended in serum‐free DMEM medium and seeded with 50,000 cells per well in the upper layer of the chamber, while the lower layer was the complete medium containing 10% FBS. When conducting cell invasion assays, the filtration membrane surface of the chamber was coated with 10% matrigel. The cultivation times for invasion and migration assays were 36 and 24 h, respectively. After cultivation, we fixed the cells with 4% paraformaldehyde and stained them with crystal violet. Finally, a conventional optical microscope was used for cell photography, and the ImageJ software was used for cell counting.

### 2.11 Irradiation

Irradiation for cells: Cultured cells in dishes or plates were placed at the center of the irradiation platform in an X‐RAD 225Cx biological irradiator (Precision X‐Ray, USA). Irradiation parameters were set to 15 mA current and 225 kV voltage, with exposure duration calibrated to achieve the prescribed radiation dose. Following irradiation, cells were returned to the incubator for continued culture.

Irradiation for murine tumors: Anesthetized mice were immobilized on a specialized irradiation stage, and tumor‐specific collimators were installed to confine radiation exposure to the tumor volume. The integrated positioning system was utilized to precisely align the isocenter of the radiation beam with the centroid of the tumor mass. A radiation dose of 6 Gy was administered at the predetermined dose rate, resulting in an exposure time of 2 min per mouse.

### 2.12 Colony Formation Assay

Cells transfected for 48 h were collected and seeded into 6‐well plates, with cell numbers of 800, 1000, 2000, 3000, and 4000, respectively, and 3 replicates per group. After 24 h, the cells were irradiated with X‐rays at doses of 0, 2, 4, 6, and 8 Gy, respectively. They were then cultured in a 37°C incubator for 10–14 days, with a new complete culture medium replaced every 3 days. When visible and appropriately sized clones grew from the cells, we terminated the culture and fixed the cells with 4% paraformaldehyde before giving them crystal violet staining. ImageJ software was used to count clones with more than 50 cells. Finally, we fitted the radiobiological multi‐target single‐hit model curve in GraphPad Prism 9 software using the formula SF = 1—(1—EXP (‐D/D0)) ^ N and calculated the sensitization enhancement ratio (SER).

### 2.13 Flow Cytometry

Cell apoptosis assay: The cells to be tested were collected and stained with Annexin V‐FITC and pyridine iodide (PI) dyes, and analyzed using flow cytometry on FITC and PE channels.

Cell ROS assay: The cells to be tested were collected and suspended in a diluted fluorescent probe (DCFH‐DA). After the probe was loaded, we used a flow cytometer to analyze the cells on the FITC channel.

### 2.14 Immunofluorescence (IF) Staining

U251 and LN229 cells were seeded onto cell slides in a 6‐well plate, then received irradiation and collected at appropriate time points. Cells were sequentially fixed with 4% paraformaldehyde, permeabilized with 0.5% TritonX‐100, and blocked with 5% BSA. The primary antibody working solution (γ‐H2AX) was incubated overnight at 4°C. After cleaning, the fluorescent secondary antibody was incubated at room temperature in the dark for 1 h. Finally, the cell slides were sealed with a solution containing DAPI. We observed and imaged cells using a Leica confocal microscope, and used ImageJ software for γ‐H2AX foci counting. The list of antibodies used in this study is provided in (Table S2).

### 2.15 Stable Glioma Cell Line Construction

Lentiviruses for GPX8 knockdown were packaged and purified by Corues Biotechnology (Nanjing, China). U87 and SHG‐44 cells were transfected with the following lentiviral vectors in the presence of Polybrene (10 μg/mL): Lentivirus/NC (shNC, negative control), Lentivirus/U6‐GPX8‐i1 (shGPX8‐1), Lentivirus/U6‐GPX8‐i2 (shGPX8‐2), Lentivirus/U6‐GPX8‐i3 (shGPX8‐3). Forty‐eight hours post‐transduction, cells underwent selection with puromycin (2 μg/mL) for 7 days to establish stable GPX8‐knockdown U87 and SHG‐44 cell lines. Knockdown efficiency was validated using RT‐qPCR and Western blotting. Target sequences for GPX8 shRNAs are provided in (Table S1).

### 2.16 Xenograft Glioma Mouse Model and Treatment

Female BALB/c‐nude mice (4–6 weeks, SPF‐grade) were subcutaneously inoculated with 1 × 10^7^ U87 or SHG‐44 cells (shNC or shGPX8 stable knockdown cells generated via lentiviral infection). Tumor growth was monitored daily, with dimensions measured every 3 days. When tumors reached approximately 100–200 mm^3^, treatment with RT was initiated, depending on the cell line (15 days post‐inoculation for U87 cells and 21 days for SHG‐44 cells). Mice were randomly assigned to four groups (*n* = 6): shNC, shGPX8, shNC+RT, shGPX8 + RT. Mice in the RT group received 6 Gy of X‐ray radiotherapy once daily for 3 consecutive days (6 Gy × 3). Mice were humanely euthanized for tumor volume measurement and other analyses on Day 30 post‐inoculation for U87 tumors or on Day 36 for SHG‐44 tumors. All procedures complied with the ethical guidelines approved by the Animal Ethics Committee of Wuhan University.

### 2.17 Statistics

Bioinformatics analysis was performed using R 4.4.1 software, and experimental data were analyzed using GraphPad Prism 9 software and presented in the form of mean ± SD. For continuous variables, two‐tailed Student's *t*‐test or Wilcoxon test was used for comparison between two groups, and one‐way ANOVA or Kruskal‐Wallis test was used for comparison between multiple groups. Chi‐square test was used for comparison between groups of categorical variables. Log‐rank test was used to calculate the *p*‐value in the Kaplan–Meier survival analysis. Pearson correlation was used to analyze the correlation of gene expression. *p* < 0.05 was considered statistically significant.

## 3 Results

### 3.1 Defining the Radiotherapy Responsiveness of Glioma Patients

**FIGURE 1:**
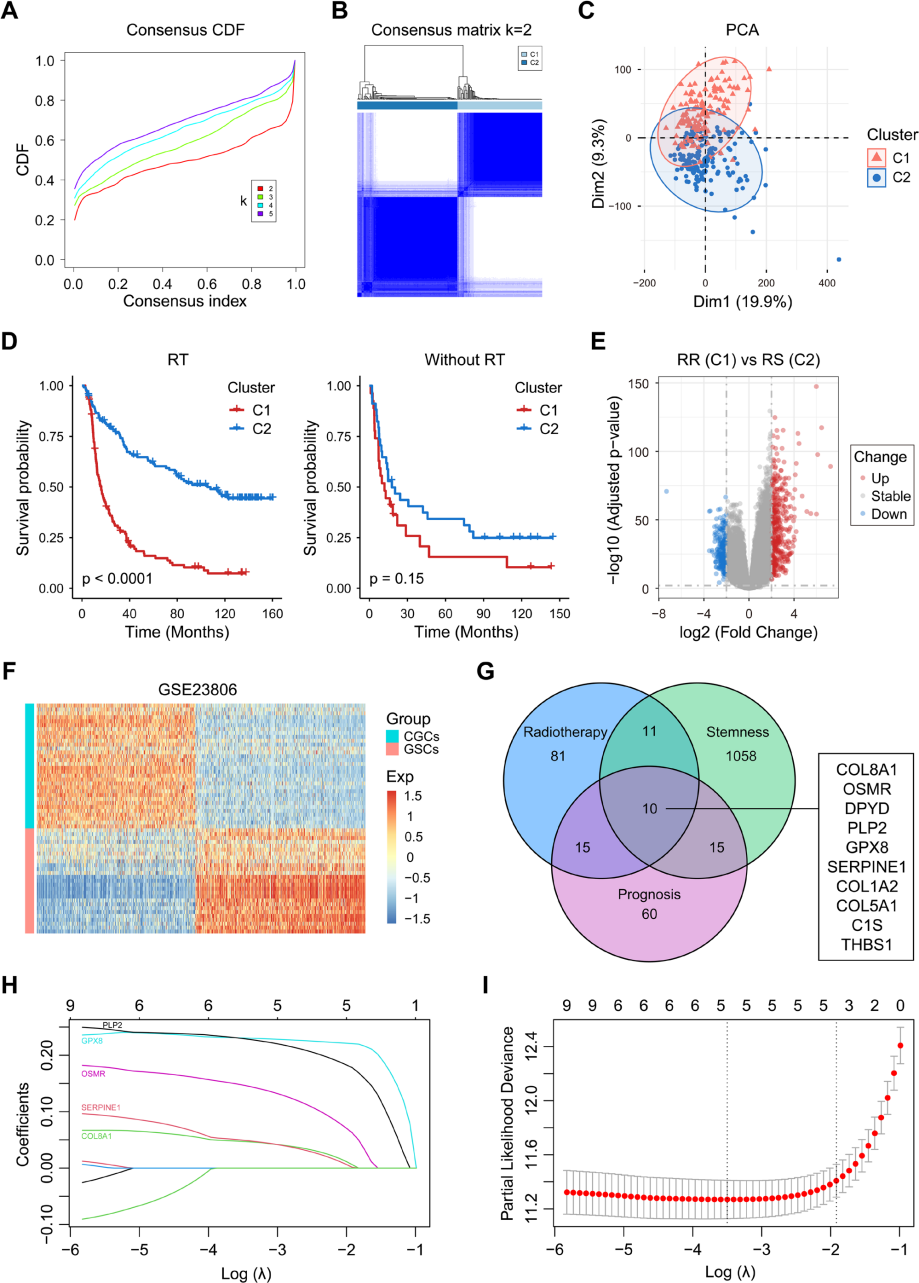
Screening of radiosensitivity‐related genes in glioma and construction of a signature. A. The consensus CDF of unsupervised consensus clustering based on 31‐gene expression profile in CGGA325 cohort. B. The consensus matrix with optimal k value (k = 2) in CGGA325 cohort. C. PCA plot of C1 and C2 groups in CGGA325 cohort. D. Kaplan–Meier survival analysis (OS) of C1 and C2 groups for patients with or without radiotherapy (RT) in CGGA325 cohort. E. Volcano plot of differential expression analysis between RR (C1) and RS (C2) group in CGGA325 cohort, with the threshold set at |log2 (fold change)| ≥ 2 and adjusted *p* < 0.01. F. Differential expression analysis of CGCs and GSCs in the GSE23806 dataset, with the same threshold setting as above. G. The intersection of genes related to radiotherapy responsiveness, tumor stemness, and prognosis in glioma. H, I. Lasso‐Cox regression on the 10 radiosensitivity‐related genes in TCGA cohort.

To identify key genes associated with radiosensitivity in glioma, we collected a 31‐gene signature summarized by Kim et al. (the authors identified target genes and pathways related to radiosensitivity by analyzing the correlation between gene expression data in four different microarray platforms and survival scores after 2Gy X‐ray irradiation [21]). Then we extracted the expression profiles of these 31 genes from two independent datasets (CGGA325 and CGGA693, patients with unknown/missing RT status and an OS of less than 30 days were excluded), and grouped the patients using an unsupervised consensus clustering algorithm. The consensus cumulative distribution function (CDF) plot (Figure 1A) showed that the clustering analysis results are most reliable when k = 2 (patients are divided into two groups, C1 and C2). In the CGGA325 cohort, there were 137 patients in C1 and 162 patients in C2 (Figure 1B). Principal component analysis (PCA) also showed good clustering performance (Figure 1C). Subsequently, through Kaplan–Meier survival analysis we found that the survival ability of C2 was significantly better than that of C1 in patients who had received radiotherapy, while there was no significant difference in survival between the two groups in patients without radiotherapy (Figure 1D). The above analysis was performed synchronously in the CGGA693 cohort and similar results were obtained (Supporting Information: Figure S1A–D). Therefore, we defined C1 with poorer prognosis as the radioresistant group (RR), and C2 with better prognosis as the radiosensitive group (RS).

### 3.2 Screening for Radiosensitivity‐Related Genes and Constructing a Signature

To obtain genes related to radiosensitivity, we performed differential expression analysis on RR and RS groups in CGGA325 and CGGA693 cohorts (threshold set as |log2 (fold change)| ≥ 2 and adjusted *p* < 0.01). A total of 679 differentially expressed genes (DEGs) were obtained in the CGGA325 cohort (Figure 1E, 411 up‐regulated and 268 down‐regulated), and 1336 DEGs were obtained in the CGGA693 cohort (Figure S1E, 808 up‐regulated and 528 down‐regulated). Subsequently, we intersected the DEGs in the two cohorts and obtained 116 target genes, which were defined as radiotherapy responsiveness related genes. Glioma stem cells typically have stronger radioresistance than conventional glioma cells (CGCs). Therefore, we performed differential expression analysis on the microarray of 27 GSCs and 32 CGCs in the GSE23806 dataset (with the same threshold settings as above), and obtained 1094 glioma stemness‐related genes (Figure 1F). Meanwhile, we utilized univariate Cox regression analysis and intersected prognostic genes in the CGGA325, CGGA693, and TCGA GBM datasets (*p* < 0.05). Finally, 10 hub genes (COL8A1, OSMR, DPYD, PLP2, GPX8, SERPINE1, COL1A1, COL5A1, C1S, THBS1) were screened out as glioma radiosensitivity‐related genes (Figure 1G).

Next, we performed Lasso‐Cox regression analysis in the TCGA cohort to narrow down 10 hub genes to 5 (PLP2, GPX8, OSMR, SERPINE1, COL8A1), found the optimal λ coefficient through cross‐validation and constructed a prognostic risk model (Figure 1H,I). The calculation of Risk‐score has been explained in the methods.

### 3.3 Validation of the Radiosensitivity‐Related Signature in Glioma

**FIGURE 2:**
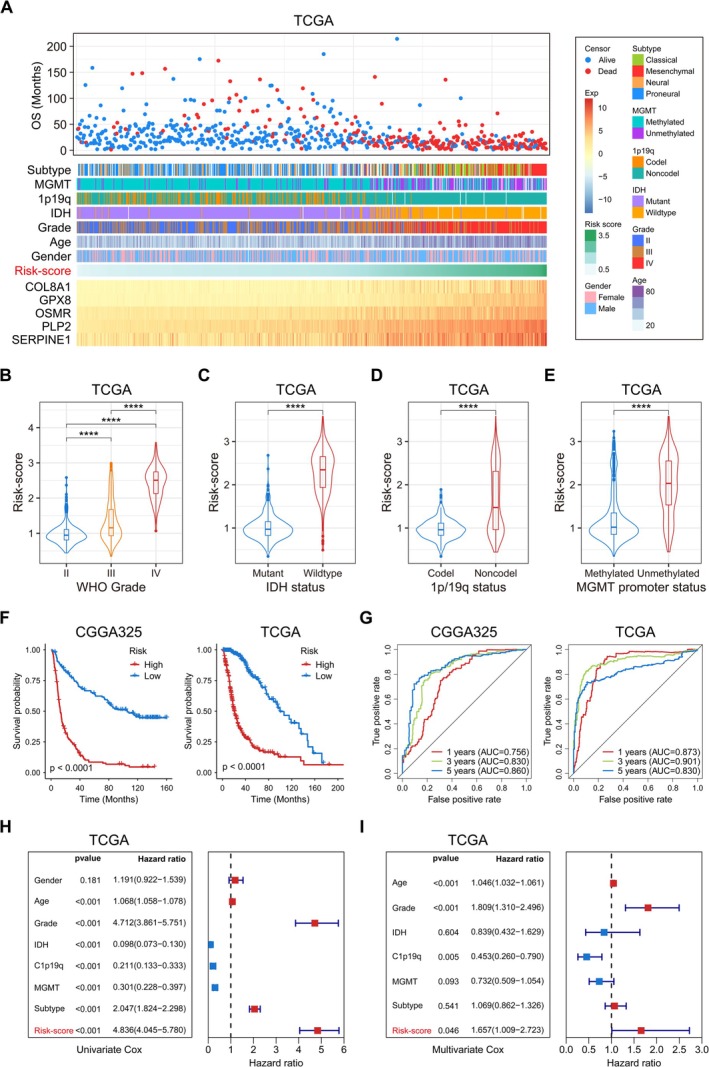
Correlation between radiosensitivity‐related signature and clinicopathological features and prognosis in glioma. A. Correlation heatmap and survival scatter plot between radiosensitivity‐related signature and clinicopathological features in TCGA cohort. B‐E. Comparison of Risk‐score for gliomas with different grades and molecular status in TCGA cohort. F. Kaplan–Meier survival analysis (OS) of radiosensitivity‐related signatures in CGGA325 or TCGA cohort. G. ROC curves (1, 3, 5 year OS) of radiosensitivity‐related signature in CGGA325 or TCGA cohort. H, I. Univariate and multivariate Cox regression analysis of OS in TCGA cohort. *p < 0.05, **p < 0.01, ***p < 0.001, ****p < 0.0001, ns means no statistical significance.

Subsequently, we validated the radiosensitivity‐related signature from two perspectives: clinicopathological features and survival prognosis. The heatmap showed the correlation between Risk‐score and clinicopathological features of glioma patients (Figure 2A, Figure S2A). Obviously, high Risk‐score was significantly enriched in high‐grade, IDH wildtype, 1p/19q non‐codeletion subtype, and MGMT promoter unmethylated gliomas (Figure 2B–E) (Figure S2B–E), which often indicate higher malignancy, lack of sensitivity to clinical treatments, and poor prognosis. Kaplan–Meier survival analysis showed that patients in the high‐risk group had poorer survival outcomes (Figure 2F), while the ROC curve demonstrated good predictive power of the signature for 1, 3, and 5 year survival abilities in glioma patients (Figure 2G). Univariate and multivariate Cox regression analysis showed that Risk‐score was an independent prognostic factor for glioma patients (Figure 2H,I, Figure S2F,G). The above results suggested that the radiosensitivity‐related signature could make accurate predictions for the malignancy and prognosis of gliomas.

### 3.4 Radiosensitivity‐Related Signature Serves as a Token of MES Glioma

**FIGURE 3:**
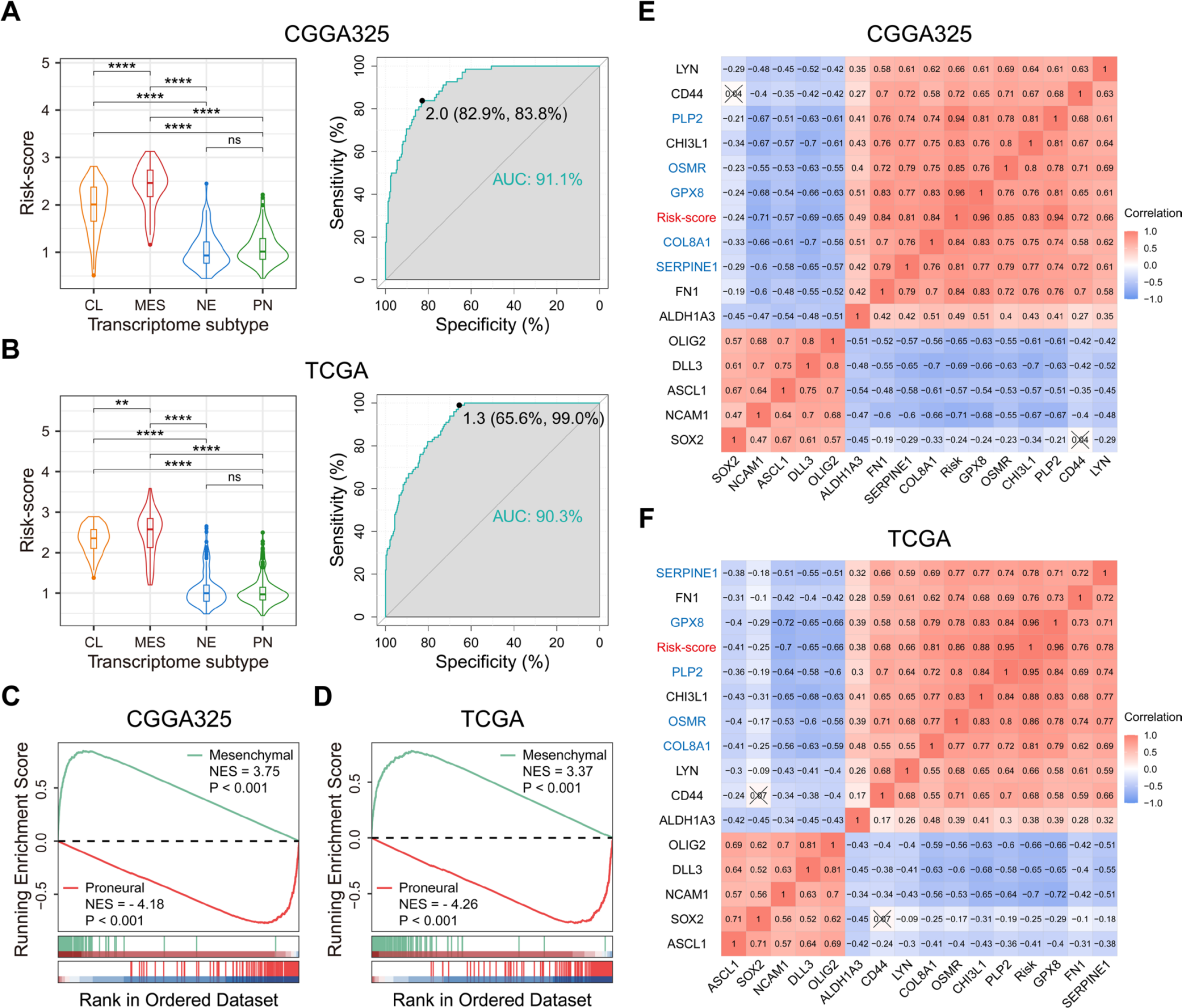
Radiosensitivity‐related signature is a token of MES glioma. A, B. The Risk‐score of MES subtype gliomas is the highest among the four transcriptome subtypes in CGGA325 or TCGA cohort, and its specificity was verified by the ROC curve. C, D. GSEA of the Risk‐score (Verhaak Glioblastoma Mesenchymal and Verhaak Glioblastoma Proneural genesets) in CGGA325 or TCGA cohort. E, F. The correlation between radiosensitivity‐related signature (Risk‐score and 5 hub genes) and MES or PN subtype glioma markers in CGGA325 or TCGA cohort (Pearson correlation analysis). *p < 0.05, **p < 0.01, ***p < 0.001, ****p < 0.0001, ns means no statistical significance.

The transcriptome typing proposed by Verhaak et al. was of great significance in determining the malignancy of gliomas and guiding clinical treatments [11]. Our study found that the Risk‐score was significantly higher in mesenchymal and classical subtype gliomas (especially the MES gliomas) than in proneural and neural subtypes. The ROC curve evaluated the specificity of Risk‐score in MES gliomas, with the area under the curve (AUC) of CGGA325 and TCGA cohorts being 91.1% and 90.3%, respectively (Figure 3A,B). The GSEA results showed a positive correlation between Risk‐score and the MES geneset, but a negative correlation with the PN geneset (Figure 3C,D). Similarly, the Risk‐score and 5 hub genes were positively correlated with typical mesenchymal subtype markers while negatively correlated with proneural subtype markers (Figure 3E,F). This suggested that the radiosensitivity‐related signature could serve as a token of MES glioma, and the hub genes might be involved in maintaining the mesenchymal subtype characteristics of malignant glioma.

### 3.5 Functional Annotation of Radiosensitivity‐Related Signature

**FIGURE 4:**
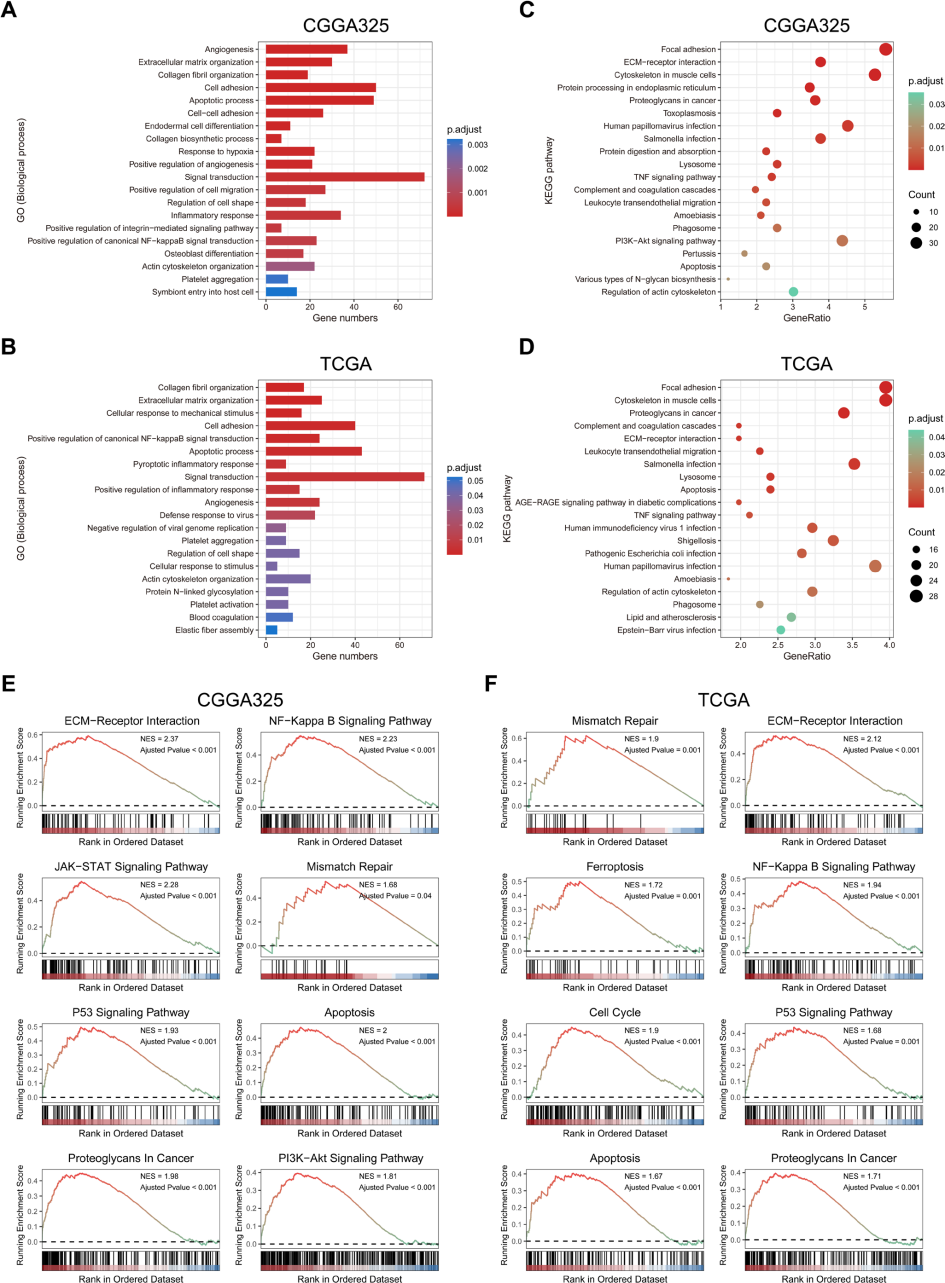
Functional annotation of radiosensitivity‐related signature in glioma. A‐D. GO (BP) and KEGG enrichment analysis of radiosensitivity‐related signature in CGGA325 or TCGA cohort. E, F. GSEA of radiosensitivity‐related signature in CGGA325 or TCGA cohort (KEGG pathway).

To understand the potential biological functions of radiosensitivity‐related signature in glioma, we submitted genes strongly correlated with the Risk‐score (Pearson r > 0.7) to the DAVID website for functional annotation. Visualized using bar charts and bubble charts were the top‑20 GO biological processes (BP) and KEGG pathways, respectively (Figure 4A–D). Furthermore, we explored the function of radiosensitivity‐related signature utilizing GSEA and demonstrated some KEGG pathways closely associated with tumor occurrence and progression. Among them, several classical signaling pathways such as p53, JAK–STAT, and NF‐κB were significantly enriched, along with those potentially affecting malignant glioma behaviors, including mismatch repair, cell cycle, and apoptosis (Figure 4E,F).

### 3.6 Radiosensitivity‐Related Signature Is Associated With the Immunosuppressive Microenvironment in Glioma

**FIGURE 5:**
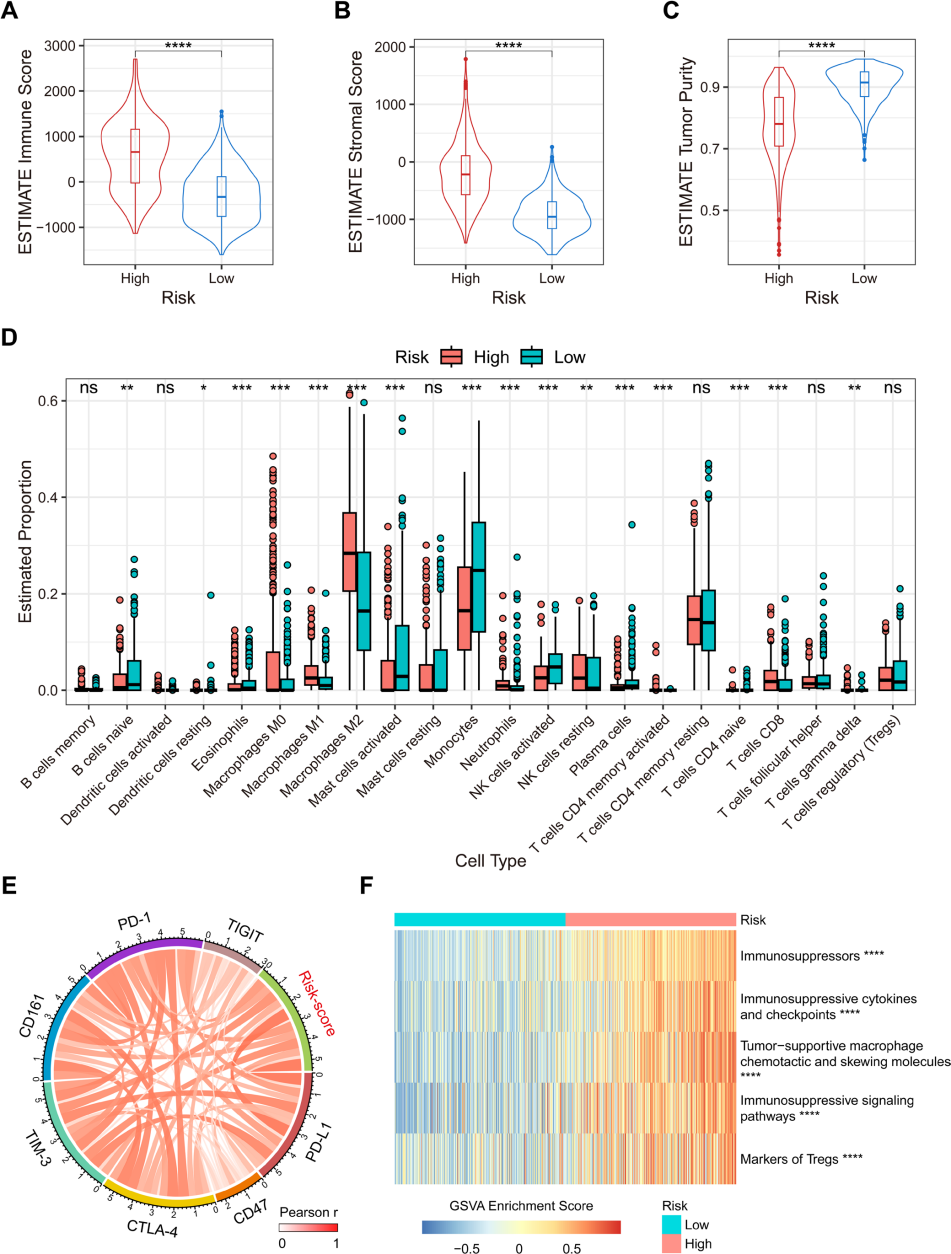
Immune‐related analysis of radiosensitivity‐related signature in glioma. A‐C. ESTIMATE analysis related to radiosensitivity‐related signature in TCGA cohort. D. Analysis of 22 types of immunocyte infiltration in glioma related to Risk‐score based on the CIBERSORT algorithm in TCGA cohort. E. The correlation between the Risk‐score and immune checkpoints expression in TCGA cohort (Pearson correlation analysis). F. GSVA of radiosensitivity‐related signature and immunosuppressive metagene in TCGA cohort. *p < 0.05, **p < 0.01, ***p < 0.001, ****p < 0.0001, ns means no statistical significance.

In the previous GSEA we enriched multiple immune‐related pathways, including antigen processing and presentation, PD‐L1 expression and PD‐1 checkpoint pathway in cancer, and natural killer cell mediated cytotoxicity (Figure S3A,B). Therefore, we speculated that the radiosensitivity‐related signature might be involved in regulating the immune microenvironment of glioma. Using the ESTIMATE algorithm, we found that high‐risk group gliomas obtained higher immune and stromal scores, while tumor purity scores were relatively lower (Figure 5A–C) (Figure S4A–C). In order to further understand the contribution of the radiosensitivity‐related signature to the tumor immune microenvironment, the infiltration of 22 types of immunocytes in glioma was evaluated based on the CIBERSORT algorithm. It is noteworthy that in CGGA325 and TCGA cohorts, the high‐risk group exhibited greater infiltration of macrophages, neutrophils, and CD8+ T cells, whereas monocytes and activated natural killer cells showed lower infiltration (Figure 5D, Figure S4D). Correlation analysis suggested that the Risk‐score was significantly positively correlated with multiple immune checkpoints (Figure 5E, Figure S4E). Next, we collected the immunosuppressive metagene [22] related to glioma as predetermined genesets for GSVA in TCGA and CGGA cohorts, including immunosuppressors, immunosuppressive cytokines and checkpoints, tumor‐supportive macrophage chemotactic and skewing molecules, immunosuppressive signaling pathways, and markers of regulatory T cells (Tregs), all of which were significantly enriched in the high‐risk group (Figure 5F, Figure S4F). It could be seen that the radiosensitivity‐related signature is associated with the immunosuppressive microenvironment in glioma.

### 3.7 GPX8 Is a Biomarker for Predicting the Malignancy and Prognosis of Glioma

**FIGURE 6:**
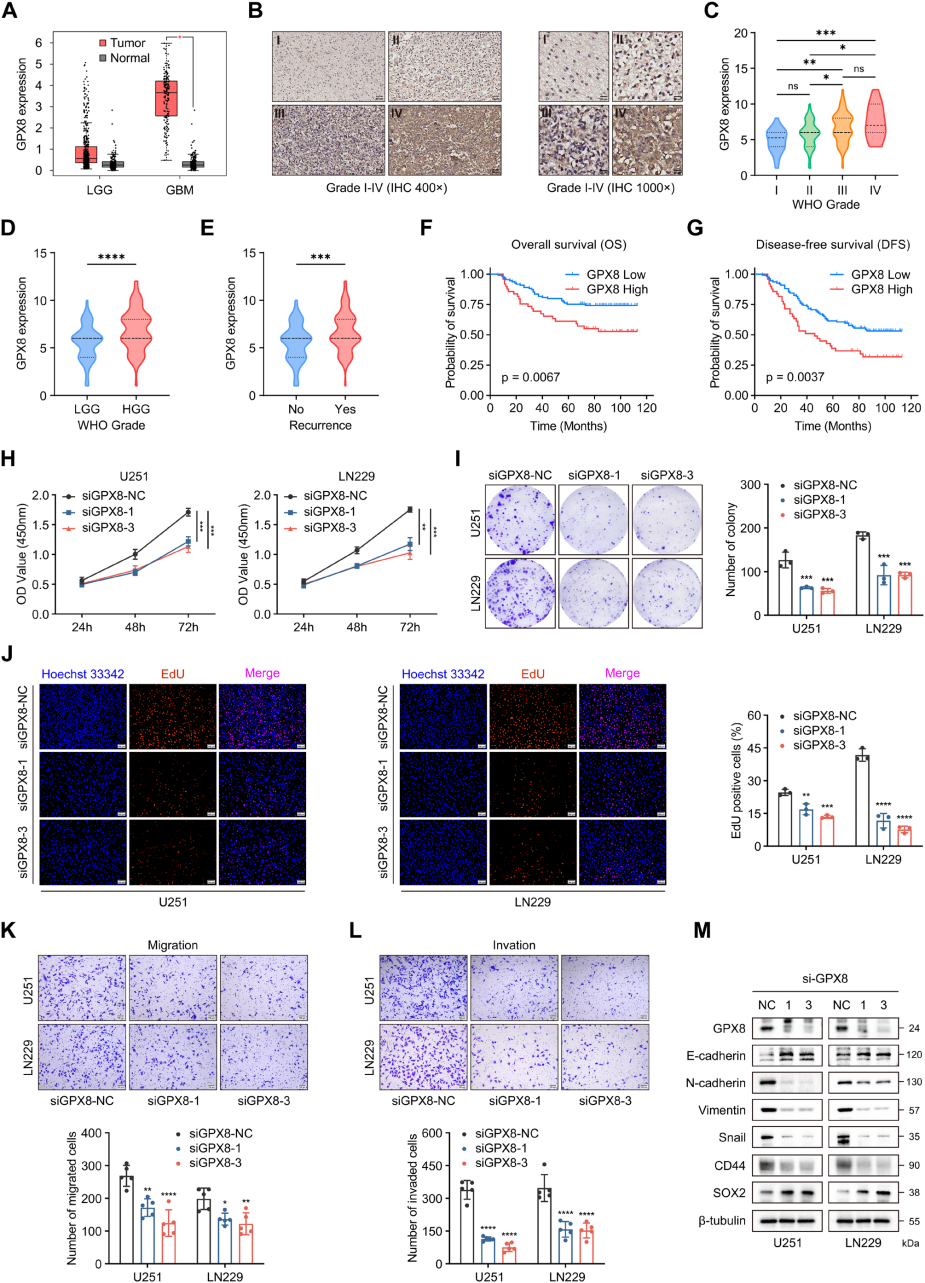
Downregulation of GPX8 inhibits the malignant biological behavior of glioma. A. The expression of GPX8 in normal brain tissue and glioma in the GEPIA database. B‐D. Expression of GPX8 in glioma tissues of different grades in the tissue microarray cohort (Immunohistochemical staining, 400×: Scale bar = 50 μm, 1000×: Scale bar = 20 μm). E. The expression of GPX8 in recurrent and non‐recurrent gliomas in the tissue microarray cohort. F, G. Kaplan–Meier survival analysis (OS and DFS) of GPX8 expression in the tissue microarray cohort. H. CCK‐8 assay for GPX8‐knockdown glioma cells. I. Clone formation assay for GPX8‐knockdown glioma cells. J. EdU assay for GPX8‐knockdown glioma cells (scale bar = 100 μm). K, L. Transwell invasion and migration assay for GPX8‐knockdown glioma cells (scale bar = 100 μm). M. The effect of knocking down GPX8 on EMT and PMT in glioma cells. *p < 0.05, **p < 0.01, ***p < 0.001, ****p < 0.0001, ns means no statistical significance.

We performed a systematic literature search on five molecules of radiosensitivity‐related signature, among which OSMR was reported to be involved in regulating mitochondrial respiration and radiosensitivity in glioma stem cells [23]. As the gene with the highest λ coefficient in the Lasso‐Cox regression, GPX8 expression contributes most substantially to the radiosensitivity‐related Risk‐score. However, the relationship between GPX8 and radiosensitivity, particularly glioma radioresistance, has not been reported. Therefore, GPX8 was selected for further investigation. In the GEPIA database, the expression of GPX8 in glioma tissue is significantly higher than that in normal brain tissue (Figure 6A). We further performed IHC staining of GPX8 on the 177‐glioma tissue microarray, and the results showed that GPX8 expression increased with the grade of glioma (Figure 6B–D). The expression of GPX8 with recurrence was significantly higher than that in non‐recurrent patients (Figure 6E), indicating that GPX8 might affect the sensitivity of postoperative treatments. By integrating and analyzing the clinical features and GPX8 expression patterns from tissue microarray patients, we observed a positive correlation between GPX8 expression and elevated age at onset (*p* = 0.001), advanced pathological grade (p = 0.001), and tumor recurrence (*p* = 0.012) (Table 1). Kaplan–Meier survival analysis of OS and disease‐free survival (DFS) showed that patients with high GPX8 expression had poor prognoses (Figure 6F,G).

**TABLE 1:** Correlation between GPX8 expression and clinical features of glioma in the tissue microarray cohort.

Features	No. of patients	GPX8 expression		*p*
		Low	High	
All patients	158	109	49	
Gender				0.473
Male	100	71	29	
Female	58	38	20	
Age				0.001
≤ 45	93	74	19	
> 45	64	35	29	
Grade				0.001
I	20	19	1	
II	73	56	17	
III	47	25	22	
IV	18	9	9	
Tumor recurrence				0.012
No	75	59	16	
Yes	83	50	33	

### 3.8 Downregulation of GPX8 Inhibits the Malignant Phenotype of Glioma Cells

We constructed three siRNAs that knocked down GPX8 in U251 and LN229 cells, and the interference efficiency was validated at both transcriptional and translational levels (Figure S5A,B). Sequences 1 and 3 (siGPX8‐1 and siGPX8‐3) were selected for subsequent experiments. The CCK‐8 assay showed that knocking down GPX8 significantly reduced the viability of GBM cells (Figure 6H) while affecting their colony‐forming ability (Figure 6I). The EdU assay more directly demonstrated that downregulating GPX8 could significantly inhibit the proliferation ability of glioma cells (Figure 6J). In the Transwell assay, we observed that U251 and LN229 showed weaker migration and invasion abilities after knocking down GPX8 (Figure 6K,L). EMT always plays a crucial role in affecting the invasion and migration ability of cancer cells as well as cancer stemness. Western blot analysis revealed that knocking down GPX8 upregulated the epithelial marker (E‐cadherin) while downregulating mesenchymal markers (N‐cadherin, Vimentin) and the key EMT transcription factor (Snail) (Figure 6M). Noteworthily, knockdown of GPX8 downregulated the MES marker CD44 and upregulated the PN marker SOX2 in U251 and LN229 cells (Figure 6M), suggesting a role for GPX8 in maintaining the mesenchymal subtype characteristics. The above results confirmed that GPX8 played a pro‐cancer role in malignant glioma.

### 3.9 Knockdown of GPX8 Enhances the Radiosensitivity of Glioma Cells

**FIGURE 7:**
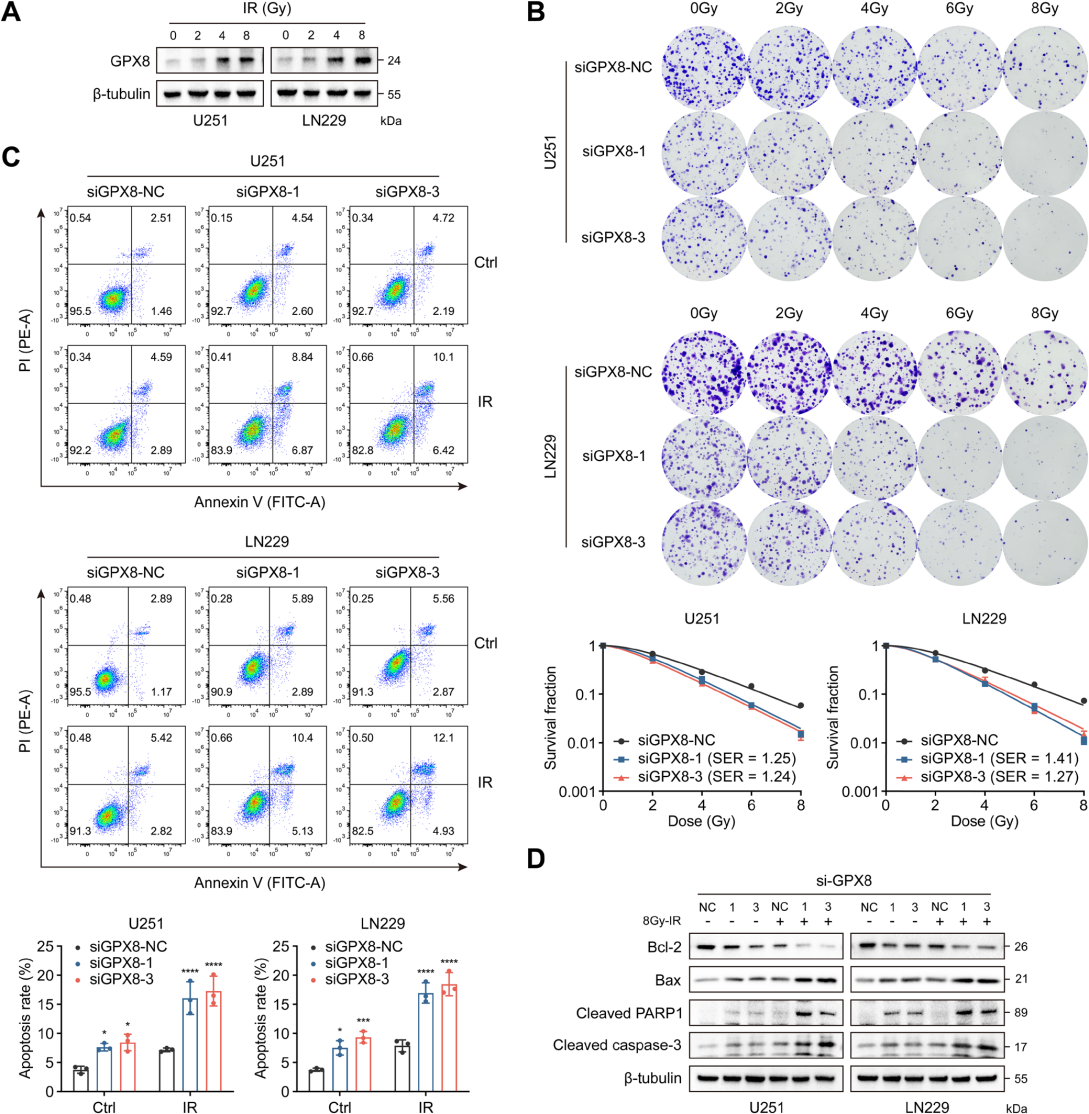
Downregulation of GPX8 enhances the radiosensitivity of glioma cells. A. The effect of radiation on GPX8 expression in glioma cells. B. Radiobiological multi‐target single‐hit model of GPX8‐knockdown glioma cells based on clone formation assay. C. Apoptosis assay for GPX8‐knockdown and/or irradiated glioma cells based on flow cytometry. D. Expression changes of apoptosis related proteins in GPX8‐knockdown and/or irradiated glioma cells. *p < 0.05, **p < 0.01, ***p < 0.001, ****p < 0.0001, ns means no statistical significance.

In the previous work, we identified GPX8 as a radiosensitivity‐related gene in glioma, therefore, the present study focuses on its impact on radiotherapy. Firstly, we exposed U251 and LN229 cells to increasing doses of irradiation (0, 2, 4, 8Gy) and found that GPX8 expression was significantly upregulated in a dose‑dependent manner (Figure 7A). These findings suggested that tumor cells may evade radiation‑induced killing by overexpressing GPX8. Therefore, we fitted the survival curves of GPX8‐knockdown glioma cells through clone formation assay following irradiation. The results showed that downregulating GPX8 significantly inhibited the survival fraction (SF) of U251 and LN229 cells at different irradiation doses. By constructing a radiobiological multi‐target single‐hit model, the sensitization enhancement ratios (SER) of two siRNAs in the U251 cell line were 1.25 and 1.24, respectively, and in the LN229 cell line were 1.41 and 1.27, respectively (Figure 7B). In addition, we observed that knocking down GPX8 increased the apoptosis rate of GBM cells, and the proportion of apoptotic cells was further increased after combined radiation (Figure 7C). Western blot analysis showed that GPX8 knockdown combined with radiation further induced apoptosis related proteins (Cleaved Caspase‐3, Cleaved PARP1, Bax) and significantly downregulated the anti‐apoptotic protein Bcl‐2 (Figure 7D). These results confirmed that inhibiting GPX8 expression enhances the radiosensitivity of glioma cells to a certain extent.

### 3.10 Downregulating GPX8 Facilitates the Radiosensitivity of Glioma via Modulation of Oxidative Stress and DNA Damage Response

**FIGURE 8:**
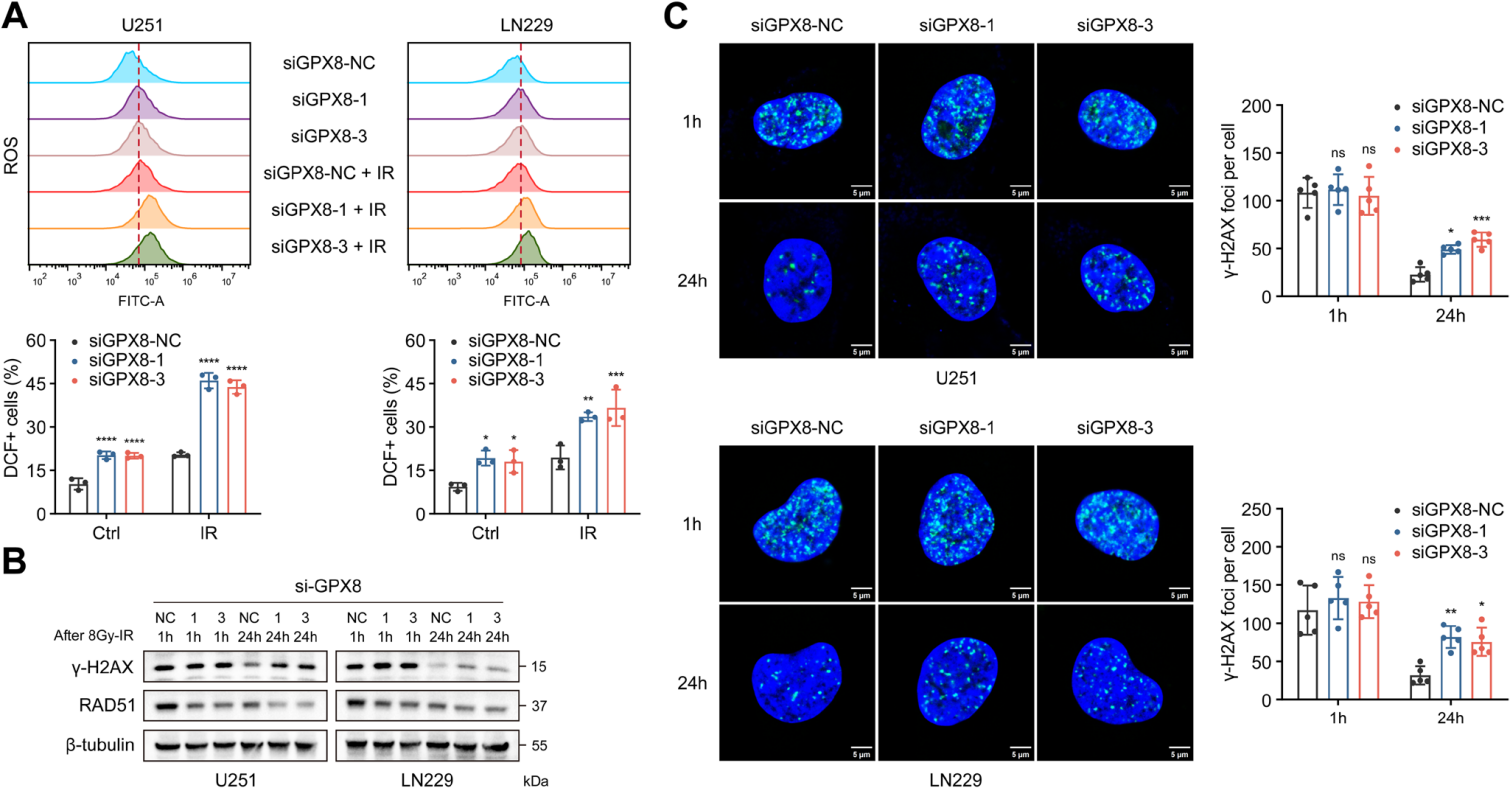
Downregulation of GPX8 enhances the radiosensitivity of glioma cells by promoting ROS accumulation and DNA damage. A. ROS assay for GPX8‐knockdown and/or irradiated glioma cells based on flow cytometry. B. Expression changes of γ‐H2AX and Rad51 in GPX8‐knockdown glioma cells at 1 or 24 h after radiation. C. Immunofluorescence staining of γ‐H2AX foci in the nucleus of GPX8‐knockdown glioma cells at 1 or 24 h after radiation (scale bar = 5 μm). *p < 0.05, **p < 0.01, ***p < 0.001, ****p < 0.0001, ns means no statistical significance.

Radiation typically kills tumor cells by inducing oxidative stress and DNA damage. Considering that GPX8 might possess glutathione peroxidase activity to scavenge oxygen free radicals, we evaluated the level of ROS in glioma cells after knocking down GPX8 using flow cytometry. It is found that inhibiting GPX8 expression significantly increased intracellular ROS levels in two cell lines, and more meaningfully, GPX8 knockdown markedly amplified radiation‐induced ROS production (Figure 8A).

When intracellular ROS cannot be cleared in time, the subsequent DNA damage pathway will be activated. γ‐H2AX is a marker of DNA double‐strand breaks and reflect the extent of DNA damage. We first evaluated the production of γ‐H2AX foci in glioma cells under 8Gy irradiation pressure. γ‐H2AX foci in U251 and LN229 cells peaked at 1 h post‐irradiation, gradually decreased, and returned to baseline levels by 24 h (Figur S5C,D). Therefore, we selected 1 h and 24 h post‐irradiation as the observation time points. In both U251 and LN229 cells, no significant difference in γ‑H2AX foci number was observed between GPX8‑knockdown cells and the control at 1 h post‑irradiation. However, at 24 h post‑irradiation, GPX8‑knockdown cells exhibited a significantly higher number of γ‑H2AX foci, which remained relatively elevated compared with the control. These findings were confirmed by immunofluorescence and Western blot analyses (Figure 8B,C). It is noteworthy that we also detected downregulation of the DNA homologous recombination repair related protein Rad51 in GPX8‐knockdown glioma cells (Figure 8B). These results indicated that GPX8 affected the radiosensitivity of glioma cells by regulating the DNA damage repair process.

### 3.11 Suppression of GPX8 Boosts the Radiosensitivity of Glioma In Vivo

**FIGURE 9:**
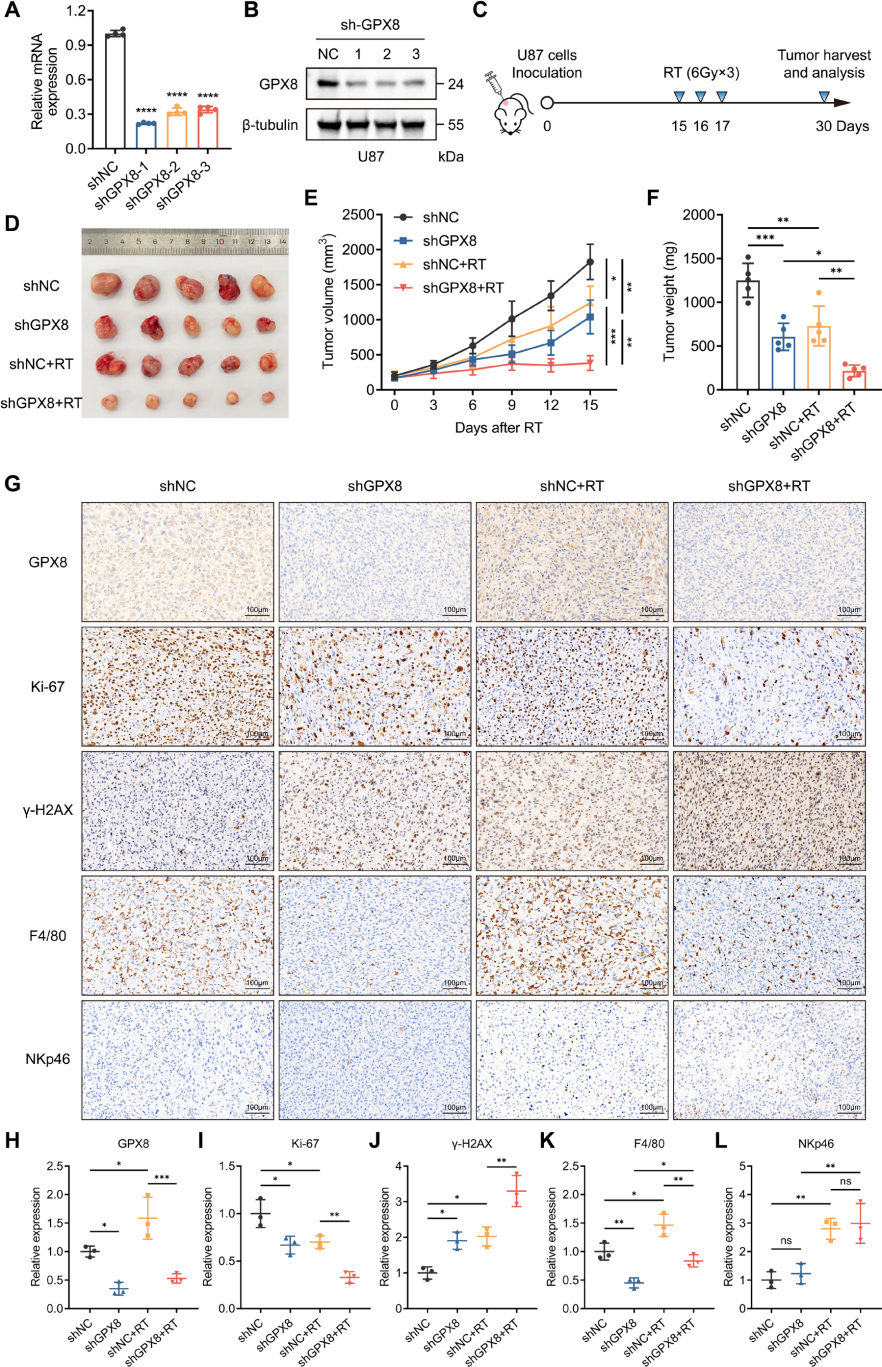
Suppression of GPX8 boosts the radiosensitivity of glioma cells in vivo. A, B. Validation of GPX8 knockdown efficiency in U87 stable cell lines generated via lentivirus‐mediated shRNA. (A) RT‐qPCR analysis. (B) Western blotting analysis. C. Experimental scheme for the U87 subcutaneous xenograft tumor model in BALB/c‐nude mice and radiotherapy treatment. D. Comparison of tumor volumes across the four experimental groups under different treatments. E. Growth curves of U87 subcutaneous xenograft tumors in the four mouse groups following radiotherapy. F. Comparison of excised tumor weights from the four treatment groups. G. Immunohistochemical (IHC) staining of mouse tumor tissues for GPX8, Ki‐67, γ‐H2AX, F4/80, and NKp46 expression (20×: Scale bar = 100 μm). H‐L. Quantitative analysis of IHC staining intensity for the respective markers. *p < 0.05, **p < 0.01, ***p < 0.001, ****p < 0.0001, ns means no statistical significance.

To further investigate whether GPX8 inhibition enhances glioma radiosensitivity in vivo, we established stable GPX8‐knockdown U87 glioma cell lines (WHO grade IV). Knockdown efficiency was validated by RT‐qPCR and Western blotting (Figure 9A,B). All three shRNA sequences effectively downregulated GPX8 expression, and the most efficient construct (shGPX8‐1) was selected for subsequent in vivo studies. Subcutaneous xenograft tumor models were generated in BALB/c nude mice using U87 cells stably expressing shNC (control) or shGPX8‐1. A subset of mice received radiotherapy (6 Gy per fraction for 3 consecutive days) on days 15–17 post‐inoculation. Mice were euthanized 2 weeks post‐radiotherapy, and tumors were harvested (Figure 9C). Tumor growth was moderately inhibited in both the shGPX8 group and the shNC+RT group compared to the shNC control group. Notably, tumor volume in the shGPX8 + RT group was significantly smaller than in all other groups (Figure 9D). Tumor growth curve monitoring and excised tumor weight comparison further confirmed a synergistic therapeutic effect between GPX8 inhibition and radiotherapy (Figure 9E,F). To extend these findings to a lower‐grade glioma model, we further evaluated the therapeutic efficacy of GPX8 knockdown combined with radiotherapy in SHG‐44 (WHO grade II‐III) subcutaneous xenografts. Consistent with the observations in U87 models, GPX8 silencing significantly enhanced the antitumor effect of radiotherapy, as evidenced by reduced tumor volume and weight in the shGPX8 + RT group compared to control or monotherapy groups (Figure S6). Collectively, these results from two distinct glioma xenograft models (WHO grade II‐III and IV) support that GPX8 inhibition potently sensitizes gliomas to radiotherapy across different pathological grades. Quantitative IHC analysis of tumor tissues revealed that radiotherapy upregulated GPX8 expression in vivo (Figure 9G,H). Furthermore, tumors in the shGPX8 + RT group exhibited the lowest expression of the proliferation marker Ki‐67 and the highest levels of the DNA damage marker γ‐H2AX (Figure 9G,I,J), corroborating our previous in vitro findings.

Prior bioinformatic analysis revealed dominant macrophage infiltration and reduced NK cell infiltration in tumors from radiosensitivity high‐risk patients. To validate this in vivo, we performed IHC staining for the macrophage marker F4/80 and the NK cell marker NKp46 on murine tumor tissues. Results demonstrated that tumors with low GPX8 expression similarly displayed reduced F4/80 staining, whereas NKp46 expression showed no significant difference compared to the control group (Figure 9G,K,L). These findings suggest that GPX8 expression specifically modulates macrophage infiltration within the tumor microenvironment. Notably, post‐radiotherapy tumor tissues showed increased infiltration of both macrophages and NK cells (Figure 9G,K,L), indicating that radiation remodels the immune microenvironment of glioma.

## 4 Discussion

Radiotherapy is one of the most important treatment measures for glioma. However, in clinical practice, a significant proportion of gliomas exhibit intrinsic radioresistance or develop acquired resistance during radiotherapy, contributing to tumor recurrence and poor prognosis [5]. In the past 20 years, scholars from various countries have carried out in‐depth exploration on the molecular mechanisms of glioma radioresistance. Among them, the production of glioma stem cells, the change of DNA damage repair ability, cell cycle arrest, the formation of hypoxic microenvironment, the induction of autophagy, the reprogramming of metabolic pathways, EMT, and other biological processes change the radiosensitivity through independent regulation or crosstalk [24, 25, 26]. Within these processes, key molecules and pathways, such as STAT3, FGFR1, CDH2, and ALDH1A3, play critical regulatory roles in radioresistance [27, 28, 29, 30]. The primary strategy for overcoming glioma radioresistance involves identifying molecular targets that regulate these biological processes and disrupting radioresistance acquisition, thereby improving radiosensitivity.

We performed deep mining of transcriptome data through machine learning, defined patient radiotherapy responsiveness based on gene expression patterns and survival prognosis differences. Tumor stemness was prioritized as a key criterion for identifying molecules potentially modulating glioma radiosensitivity. Notably, OSMR has been implicated in regulating mitochondrial respiration and radiosensitivity in glioma stem cells [23], while SERPINE1 is a key regulator of obesity‐mediated radioresistance in triple‐negative breast cancer [31]. This independent evidence supports the biological plausibility of our radiosensitivity‐associated molecules. Furthermore, the radiosensitivity‐related signature we developed demonstrates robust validation across independent datasets and exhibits high predictive accuracy for tumor malignancy and patient outcomes. Future studies could also integrate direct radiation‐response gene expression profiles from in vitro or in vivo models to further dissect the dynamic molecular changes associated with acquired radioresistance.

The transcriptome‐based classification proposed by Verhaak et al. significantly advanced the shift from traditional pathological subtyping to molecular subtyping in malignant glioma [11]. Multiple studies indicate that gliomas of the mesenchymal and classical subtypes are more malignant than those of the proneural and neural subtypes. Notably, MES and PN subtypes, representing two extremes, exhibit striking differences in treatment responsiveness and patient survival outcomes [12, 32]. Studies have also reported distinct radiosensitivity profiles between these subtypes, with regulation by core molecules and associated pathways appearing central to this difference [27, 33, 34]. This study establishes a close association between the radiosensitivity‐related signature and the mesenchymal glioma subtype. The signature composed of five key radiosensitivity‐related molecules could accurately predict MES subtype gliomas, and they were highly likely to be involved in the transformation of PN‐MES phenotype and maintenance of MES status in malignant gliomas. In previous studies, OSMR and SERPINE1, the key molecules in our signature, have not only been proven to be biomarkers for MES subtype gliomas but also as regulators of radiosensitivity in malignant tumors [35, 36]. Consistently, a recent study by Li et al. demonstrated that GPX8 was significantly upregulated in GBM and correlated with the MES subtype, suggesting its potential involvement in proneural‐mesenchymal transition (PMT) [37]. Similarly, we have experimentally confirmed the regulatory role of GPX8 in PMT and radiosensitivity of glioma, providing direct functional evidence that extends beyond the correlative findings of previous reports. It can be seen that acquisition of the MES phenotype is mechanistically linked to the development of glioma radioresistance.

Our study further implicates the radiosensitivity‐related signature in shaping the immunosuppressive niche of glioma. Gliomas with high Risk‐score exhibited greater immunocyte infiltration and significantly enriched extracellular matrix (ECM) components, establishing a structural foundation for immunosuppression. Specifically, the Risk‐score correlated positively with macrophage and neutrophil infiltration but negatively with monocyte and NK cell infiltration. Multiple studies have shown that glioma‐associated macrophages and neutrophils were more likely to play a supportive role in maintaining tumor cell growth and immune escape [38, 39], while infiltration of monocytes and natural killer cells was associated with better survival prognosis [40, 41]. Notably, while CD8+ T cells with antitumor potential were abundant in high‐risk gliomas, the positive correlation between immune checkpoints and Risk‐score, as well as the upregulation of immunosuppressive pathways, suggested that the radiosensitivity‐related signature is at least partially involved in the generation and maintenance of the tumor immunosuppressive microenvironment.

As the primary contributor within the signature, GPX8 expression carries the highest weightage in the radiosensitivity‐related Risk‐score calculation. However, the relationship between GPX8 and radiosensitivity, particularly in the context of glioma radioresistance, has not been reported. Therefore, we conducted a more in‐depth study on GPX8. GPX family genes typically function in intracellular oxidative stress responses. Whereas GPX8 exhibits low glutathione‐dependent peroxidase activity due to the absence of a glutathione‐binding domain, and its function was mainly related to oxidative protein folding mediated by protein disulfide isomerase (PDI) peroxides [42]. On the contrary, subsequent studies have shown that GPX activity of GPX8 can be induced under endoplasmic reticulum (ER) stress conditions, thereby preventing H_2_O_2_ leakage from ER [43]. In addition, Mehmeti et al. confirmed that selective expression of GPX7 or GPX8 could enhance the ER antioxidant capacity of rat pancreatic β cells without affecting insulin production and the folding mechanism of oxidative proteins [44]. It can be seen that the mechanism by which GPX8 functions still needs further exploration. In recent years, research on GPX8 in malignant tumors has gradually increased. For instance, in lung cancer and breast cancer, it affected the invasion and migration of cancer cells by regulating EMT [15, 16], which was also confirmed in our study. Mechanistically, PMT in glioma is analogous to EMT in epithelial‑derived tumors. While EMT does occur in gliomas, it appears to be functionally intertwined with PMT processes. In addition, GPX8 was also involved in regulating lipid metabolism processes in renal clear cell carcinoma [18], calcium flow in cervical cancer cells [45], and apoptosis and autophagy in esophageal cancer cells [17], which were precisely related to the acquisition of radioresistance by tumor cells. With respect to glioma, several recent studies have investigated the expression and functional roles of GPX8. Using an ENU induced rat brain tumor model, Cueto‐Ureña et al. observed elevated expression of Gpx8 in tumor tissues compared to normal brain, highlighting its potential involvement in brain tumor biology [46]. Meanwhile, Ren et al. performed a pan‐cancer analysis and identified GPX8 as a valuable diagnostic biomarker in multiple cancers, including GBM and LGG; they further confirmed that GPX8 was upregulated in GBM cells and contributed to migration and invasion [47]. These studies collectively established the oncogenic role of GPX8 in glioma. However, neither study explored the relationship between GPX8 and radiotherapy response, a critical determinant of glioma prognosis.

Malignant gliomas, particularly high‐grade variants, characteristically display high proliferative activity and aggressive invasiveness. We have validated the expression of GPX8 and its prognostic significance in gliomas of different grades by use of tissue microarray technology. Subsequent in vitro functional experiments demonstrated that knockdown of GPX8 inhibited the proliferation, invasion, and migration abilities of glioma cells, as well as the EMT and PMT processes. Consistent with our findings, Yang et al. reported that GPX8 knockdown in U87MG and U118MG cells led to G1 cell cycle arrest, increased cell death, and reduced colony formation, supporting the role of GPX8 in glioma cell proliferation and survival [48]. These findings indicated the momentous role of GPX8 in the malignant progression of glioma.

Most critically, while previous studies on GPX8 in glioma primarily focused on its prognostic significance, expression patterns, and impact on malignant phenotypes [37, 47], the present study is the first to establish its functional role in regulating radiosensitivity. Through radiobiological experiments and murine xenograft models, we found that radiation induced GPX8 expression in a dose‐dependent manner, whereas GPX8 knockdown synergistically enhanced radiation cytotoxicity by promoting oxidative stress and activating DNA damage. Ionizing radiation induces DNA damage to varying degrees when accumulated ROS overwhelm cellular clearance mechanisms. Irreparable DNA lesions cause genomic instability, triggering cell cycle arrest and ultimately activating apoptosis in tumor cells [49]. Therefore, timely clearance of intracellular ROS is essential for protecting cells from ionizing radiation‐induced damage. As a member of the glutathione peroxidase family, GPX8 may therefore regulate radiosensitivity through its antioxidant activity. Notably, we also observed that GPX8 knockdown affected the expression of the homologous recombination repair protein Rad51, suggesting that the role of GPX8 in the dynamic process of DNA damage repair following oxidative stress warrants further investigation. Using two distinct xenograft models (WHO grade II‐III and IV), we further confirmed that GPX8 inhibition potentiates radiotherapy efficacy in vivo. Collectively, these findings bridge the established oncogenic functions of GPX8 with its previously unrecognized role in radioresistance, positioning GPX8 as a dual‐functional target for suppressing malignant progression and overcoming radioresistance in glioma.

In the in vivo phase of this study, we also observed a positive correlation between GPX8 expression and tumor‐associated macrophage (TAM) infiltration. TAMs in glioma typically include macrophages recruited from the peripheral circulation and CNS‐intrinsic, tissue‐resident microglia. Although distinct in origin, both populations are pivotal in shaping the highly immunosuppressive and pro‐tumorigenic microenvironment of glioma. They drive tumor growth, invasion, angiogenesis, stem cell maintenance, and therapy resistance through multiple mechanisms [50]. Lower TAM infiltration was observed in GPX8‐inhibited glioma tissues, suggesting that targeting GPX8 may boost radiotherapy efficacy not only by directly amplifying radiation cytotoxicity, but also potentially by remodeling the immune microenvironment. Future research could focus on combination therapeutic strategies targeting GPX8, radiotherapy, and immunotherapy. However, we observed no significant impact of GPX8 inhibition on NK cell infiltration, a finding potentially limited by the low baseline abundance of NK cells within the tumor tissue and the incomplete immune system of the nude mouse model. Furthermore, radiotherapy promoted increased infiltration of both macrophages and NK cells in glioma tissues, highlighting its dual role in remodeling the tumor immune microenvironment [51]. Given the immunocompromised status of nude mice and the failure of subcutaneous xenograft models to recapitulate the clinical relevance of intracranial gliomas, particularly the presence of the blood–brain barrier, region‐specific immune cells (e.g., microglia), and interactions with neural and glial components. These limitations may affect the translational relevance of our therapeutic findings. Therefore, establishing immunocompetent animal models along with orthotopic glioma models is essential for investigating how GPX8 targeting enhances radiotherapy efficacy and safety while reversing the immunosuppressive microenvironment.

## 5 Conclusions

This study identified key radiosensitivity‐associated molecules and constructed a prognostic risk model integrating radiotherapy responsiveness and glioma stemness. This model demonstrates significant potential for predicting clinical outcomes and patient survival. Furthermore, the signature serves as a biomarker for the mesenchymal subtype and correlates positively with an immunosuppressive microenvironment. Experimentally, GPX8 knockdown inhibited glioma cell proliferation, invasion, and migration while significantly enhancing radiation cytotoxicity. This radiosensitization occurred through amplified oxidative stress and DNA damage, ultimately inducing apoptosis. Suppression of GPX8 effectively potentiated radiosensitivity in murine xenograft models and reduced intratumoral infiltration of tumor‐associated macrophages. Collectively, GPX8 emerges as a pivotal regulator of glioma malignancy and radiosensitivity, representing a promising therapeutic target for countering tumor progression and radioresistance.

## Author Contributions

Gan Tao, Yahua Zhong and Qiuji Wu conceptualized this study; Gan Tao performed experimental operations and data analysis, prepared the figures, and wrote the main manuscript; Xiuli Guo, Fajian He and Qiuji Wu participated in functional experiments and figure preparation; Xiaowan Guo and Kehua Hu assisted in the data analysis; Xiuli Guo, Sisi Yan, Conghua Xie and Yahua Zhong helped revise the manuscript and figures; Yahua Zhong sponsored, conceived of, and supervised the study. All authors have read and agreed to the published version of the manuscript.

## Funding

This work was supported by the Health Commission of Hubei Province Scientific Research Project (grant no. WJ2023M068) and the Leading Discipline of Oncology Construction Project of Zhongnan Hospital of Wuhan University (grant no. XKJS202005).

## Ethics Statement

The tissue microarray containing tumor samples from 177 glioma patients was purchased from Xinchao Biotechnology (Shanghai, China). All patients have signed informed consent forms and were approved by the company's ethics committee (YB M‐05‐02). In vivo experiments were conducted following the guidelines and approval from the Animal Ethics Committee of Wuhan University (ZN2025178).

## Conflicts of Interest

The authors declare no conflicts of interest.

## Supporting information


**Figure S1:** Screening of radiosensitivity‐related genes in glioma. A. The consensus CDF of unsupervised consensus clustering based on 31‐genes expression profile in CGGA693 cohort. B. The consensus matrix with optimal k value (k = 2) in CGGA693 cohort. C. PCA plot of C1 and C2 Groups in CGGA693 cohort. D. Kaplan–Meier survival analysis (OS) of C1 and C2 groups for patients with or without radiotherapy in CGGA693 cohort. E. Volcano plot of differential expression analysis between RR (C1) and RS (C2) group in CGGA693 cohort, with the threshold set at |log2 (fold change)| ≥ 2 and adjusted *p* < 0.01.
**Figure S2:** Correlation between radiosensitivity‐related signature and clinicopathological features and prognosis in glioma. A. Correlation heatmap and survival scatter plot between radiosensitivity‐related signature and clinicopathological features in CGGA325 cohort. B‐E. Comparison of Risk‐score for gliomas with different grades and molecular status in CGGA cohort. F, G. Univariate and multivariate Cox regression analysis of OS in CGGA325 cohort. *p < 0.05, **p < 0.01, ***p < 0.001, ****p < 0.0001, ns means no statistical significance.
**Figure S3:** The correlation between radiosensitivity‐related signature and immune related pathways. A, B. GSEA (KEGG pathway) of radiosensitivity‐related signature for immune related pathways in CGGA325 or TCGA cohort.
**Figure S4:** Immune related analysis of radiosensitivity‐related signature in glioma. A‐C. ESTIMATE analysis related to radiosensitivity‐related signature in CGGA cohort. D. Analysis of 22 types of immunocyte infiltration in glioma related to Risk‐score based on the CIBERSORT algorithm in CGGA cohort. E. The correlation between the Risk‐score and immune checkpoints expression in CGGA cohort (Pearson correlation analysis). F. GSVA of radiosensitivity‐related signature and immunosuppressive metagene in CGGA cohort. *p < 0.05, **p < 0.01, ***p < 0.001, ****p < 0.0001, ns means no statistical significance.
**Figure S5:** A, B. Efficiency validation of knocking down GPX8 in U251 and LN229 cells using siRNAs. C, D. The level of γ‐H2AX foci production in the nucleus of glioma cells at different time points after receiving 8Gy X‐ray irradiation (scale bar = 5 μm). *p < 0.05, **p < 0.01, ***p < 0.001, ****p < 0.0001, ns means no statistical significance.
**Figure S6:** Suppression of GPX8 boosts the radiosensitivity of glioma cells in vivo. A, B. Validation of GPX8 knockdown efficiency in SHG‐44 stable cell lines generated via lentivirus‐mediated shRNA. (A) RT‐qPCR analysis. (B) Western blotting analysis. C. Experimental scheme for the SHG‐44 subcutaneous xenograft tumor model in BALB/c‐nude mice and radiotherapy treatment. D. Comparison of tumor volumes across the four experimental groups under different treatments. E. Growth curves of SHG‐44 subcutaneous xenograft tumors in the four mouse groups following radiotherapy. F. Comparison of excised tumor weights from the four treatment groups.
**Table S1:** Sequence of primers, siRNAs and shRNAs (5 to 3).
**Table S2:** Antibodies used in this study.

## Data Availability

The data that support the findings of this study are available on request from the corresponding author. The data are not publicly available due to privacy or ethical restrictions.
