# Supplementary material for: Identification of a Novel Radiosensitivity‐Related Signature and Validation of GPX8 in Regulating the Radiosensitivity of Glioma
Source: CNS Neurosci Ther. 2026 Apr 28;32(4):e70900. doi: 10.1002/cns.70900 (PMC13123455; doi:10.1002/cns.70900)
Supplement: Supplementary file 1 — Figure S1: Screening of radiosensitivity‐related genes in glioma. A. The consensus CDF of unsupervised consensus clustering based on 31‐genes expression profile in CGGA693 cohort. B. The consensus matrix with optimal k value (k = 2) in CGGA693 cohort. C. PCA plot of C1 and C2 Groups in CGGA693 cohort. D. Kaplan–Meier survival analysis (OS) of C1 and C2 groups for patients with or without radiotherapy in CGGA693 cohort. E. Volcano plot of differential expression analysis between RR (C1) and RS (C2) group in CGGA693 cohort, with the threshold set at |log2 (fold change)| ≥ 2 and adjusted p < 0.01. Figure S2: Correlation between radiosensitivity‐related signature and clinicopathological features and prognosis in glioma. A. Correlation heatmap and survival scatter plot between radiosensitivity‐related signature and clinicopathological features in CGGA325 cohort. B‐E. Comparison of Risk‐score for gliomas with different grades and molecular status in CGGA cohort. F, G. Univariate and multivariate Cox regression analysis of OS in CGGA325 cohort. *p < 0.05, **p < 0.01, ***p < 0.001, ****p < 0.0001, ns means no statistical significance. Figure S3: The correlation between radiosensitivity‐related signature and immune related pathways. A, B. GSEA (KEGG pathway) of radiosensitivity‐related signature for immune related pathways in CGGA325 or TCGA cohort. Figure S4: Immune related analysis of radiosensitivity‐related signature in glioma. A‐C. ESTIMATE analysis related to radiosensitivity‐related signature in CGGA cohort. D. Analysis of 22 types of immunocyte infiltration in glioma related to Risk‐score based on the CIBERSORT algorithm in CGGA cohort. E. The correlation between the Risk‐score and immune checkpoints expression in CGGA cohort (Pearson correlation analysis). F. GSVA of radiosensitivity‐related signature and immunosuppressive metagene in CGGA cohort. *p < 0.05, **p < 0.01, ***p < 0.001, ****p < 0.0001, ns means no statistical significance. Figure S5: A, B. Efficiency v [file CNS-32-e70900-s001.docx]

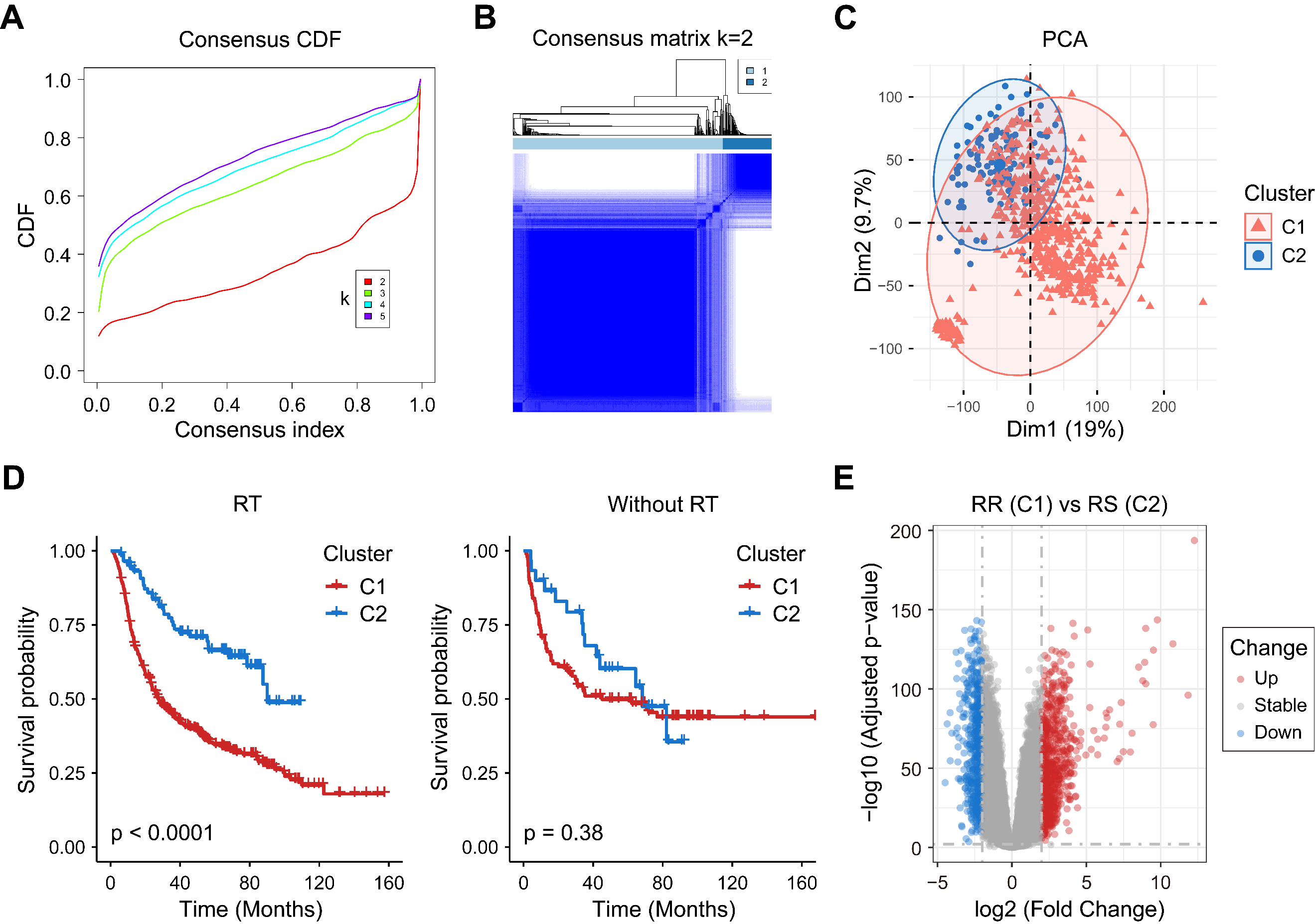
**Fig. S1. Screening of radiosensitivity-related genes in glioma.** **A.** The consensus CDF of unsupervised consensus clustering based on 31-genes expression profile in CGGA693 cohort. **B.** The consensus matrix with optimal k value (k = 2) in CGGA693 cohort. **C.** PCA plot of C1 and C2 Groups in CGGA693 cohort. **D.** Kaplan-Meier survival analysis (OS) of C1 and C2 groups for patients with or without radiotherapy in CGGA693 cohort. **E.** Volcano plot of differential expression analysis between RR (C1) and RS (C2) group in CGGA693 cohort, with the threshold set at |log2(fold change)| ≥ 2 and adjusted p-value < 0.01.


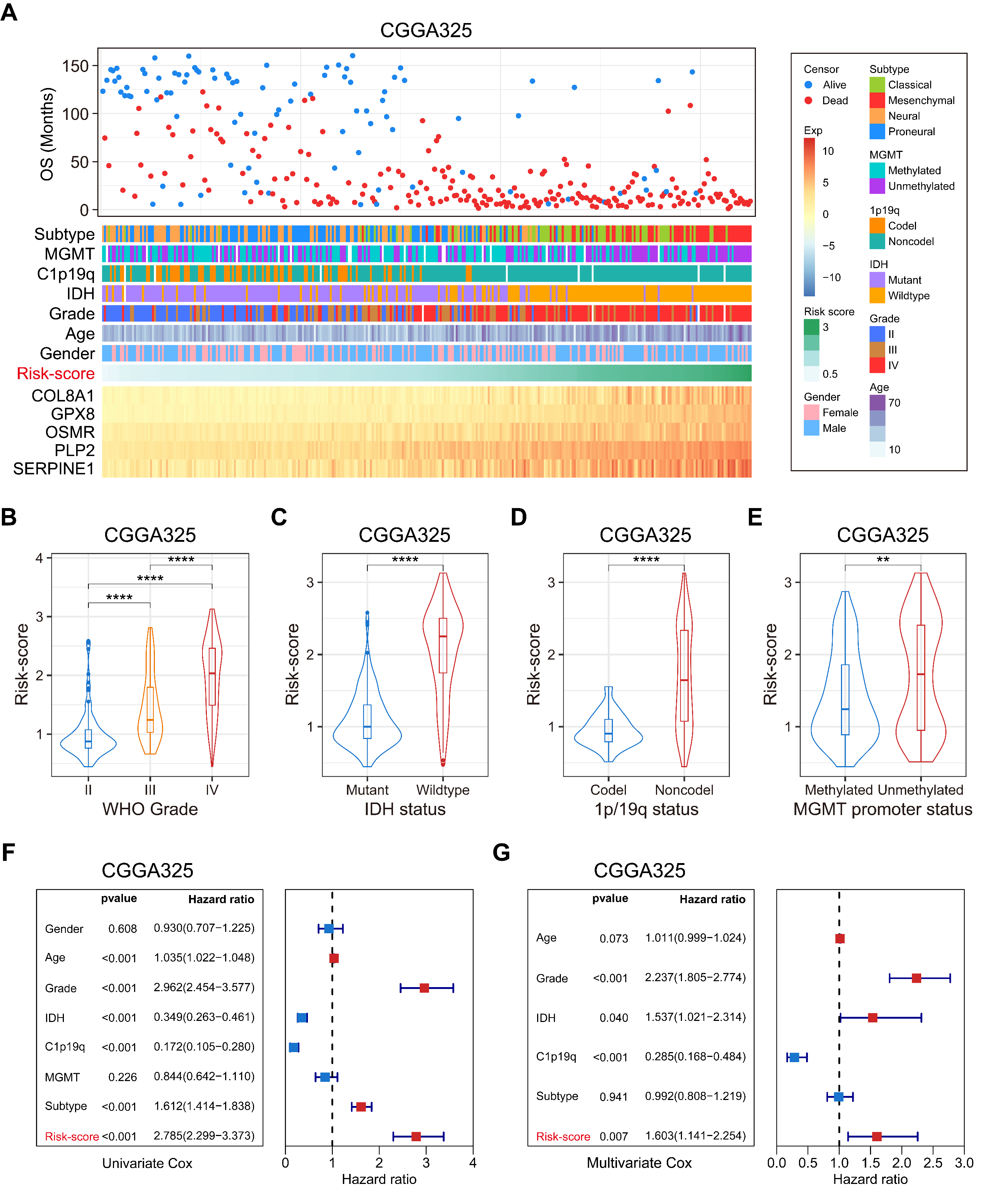
**Fig. S2. Correlation between radiosensitivity-related signature and clinicopathological features and prognosis in glioma. A.** Correlation heatmap and survival scatter plot between radiosensitivity-related signature and clinicopathological features in CGGA325 cohort. **B-E.** Comparison of Risk-score for gliomas with different grades and molecular status in CGGA cohort. **F, G.** Univariate and multivariate Cox regression analysis of OS in CGGA325 cohort. *p < 0.05, **p < 0.01, ***p < 0.001, ****p < 0.0001, ns means no statistical significance.


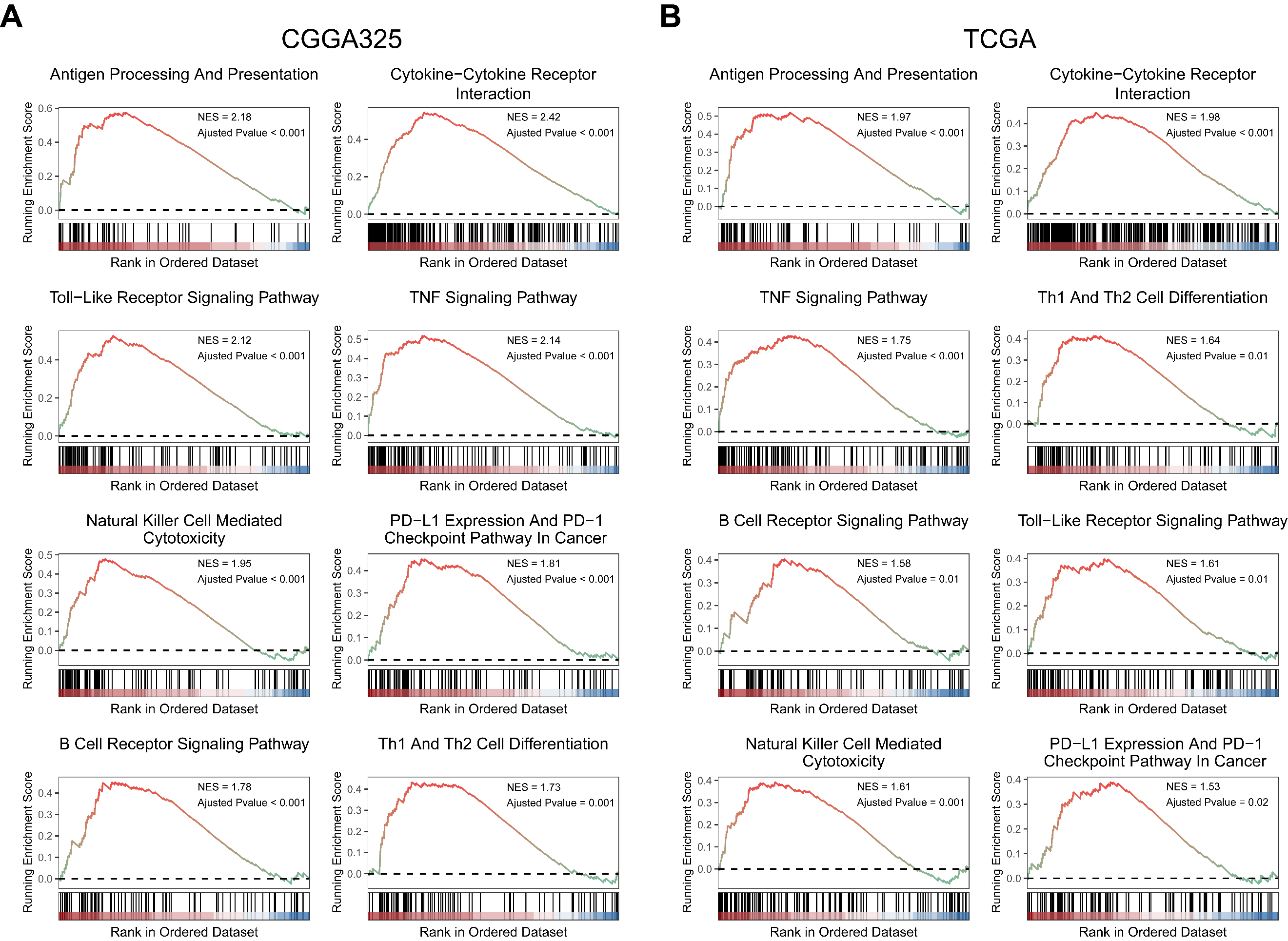
**Fig. S3. The correlation between** **radiosensitivity-related signature and immune related pathways. A, B.** GSEA (KEGG pathway) of radiosensitivity-related signature for immune related pathways in CGGA325 or TCGA cohort.

**
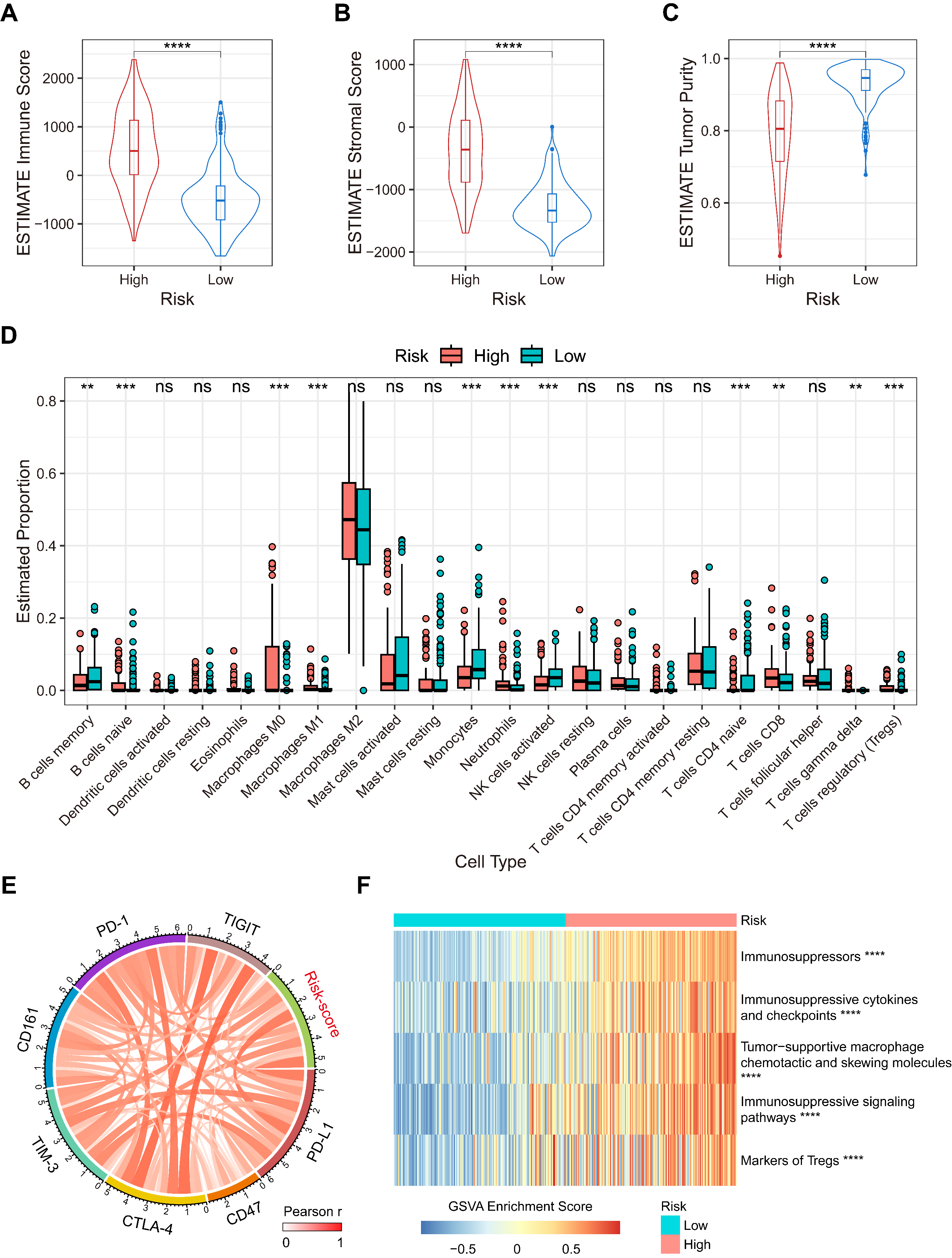
Fig. S4. Immune related analysis of radiosensitivity-related signature in glioma. A-C.** ESTIMATE analysis related to radiosensitivity-related signature in CGGA cohort. **D.** Analysis of 22 types of immunocyte infiltration in glioma related to Risk-score based on the CIBERSORT algorithm in CGGA cohort. **E.** The correlation between the Risk-score and immune checkpoints expression in CGGA cohort (Pearson correlation analysis). **F.** GSVA of radiosensitivity-related signature and immunosuppressive metagene in CGGA cohort. *p < 0.05, **p < 0.01, ***p < 0.001, ****p < 0.0001, ns means no statistical significance.


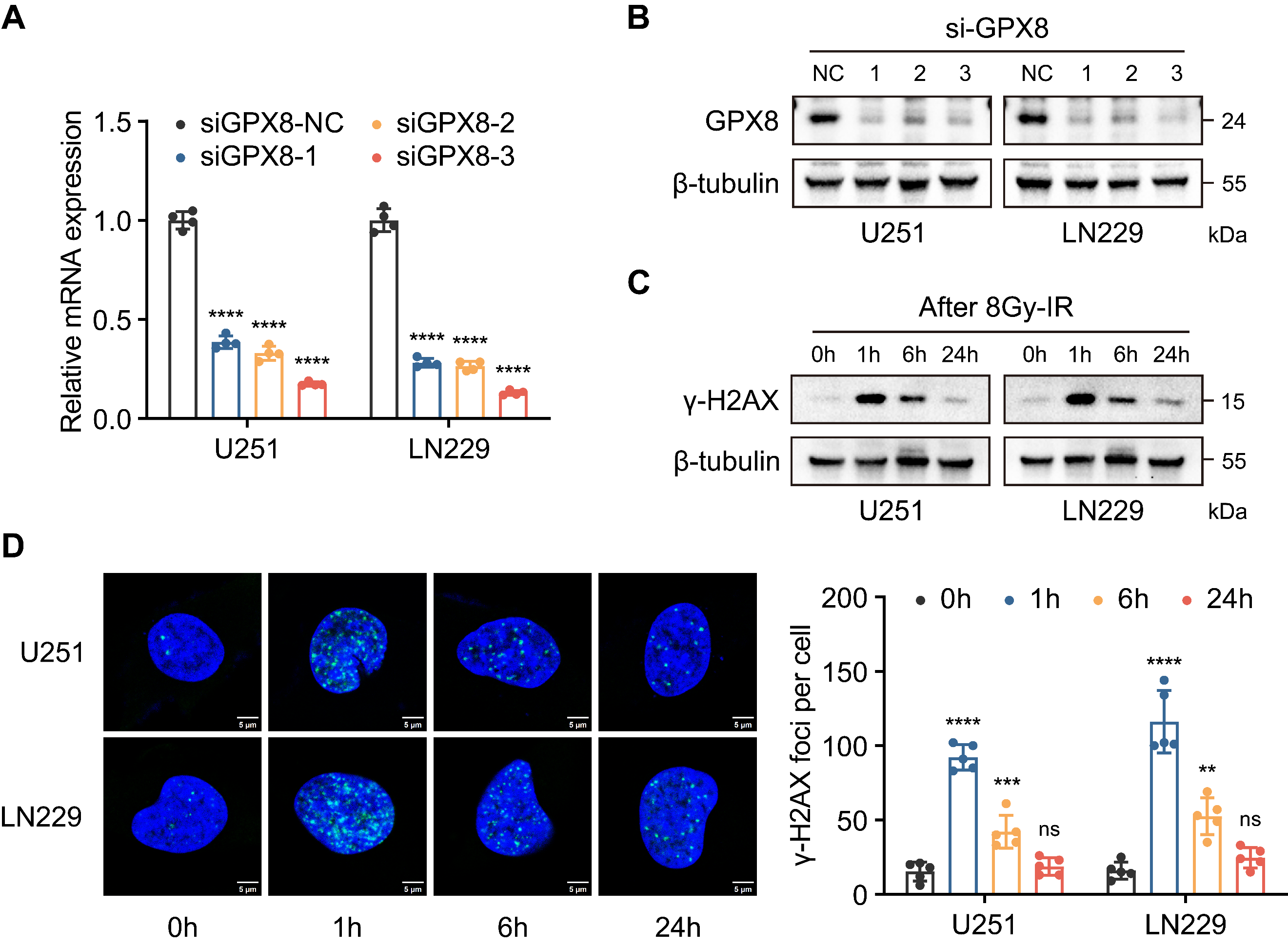


**Fig. S5. A, B.** Efficiency validation of knocking down GPX8 in U251 and LN229 cells using siRNAs. **C, D.** The level of γ-H2AX foci production in the nucleus of glioma cells at different time points after receiving 8Gy X-ray irradiation (scale bar = 5μm). *p < 0.05, **p < 0.01, ***p < 0.001, ****p < 0.0001, ns means no statistical significance.

**Fig. S6.** **Suppression of GPX8 boosts the radiosensitivity of glioma cells in vivo. A, B.** Validation of GPX8 knockdown efficiency in SHG-44 stable cell lines generated via lentivirus-mediated shRNA. (A) RT-qPCR analysis. (B) Western blotting analysis. **C.** Experimental scheme for the SHG-44 subcutaneous xenograft tumor model in BALB/c-nude mice and radiotherapy treatment. **D.** Comparison of tumor volumes across the four experimental groups under different treatments. **E.** Growth curves of SHG-44 subcutaneous xenograft tumors in the four mouse groups following radiotherapy. **F.** Comparison of excised tumor weights from the four treatment groups.
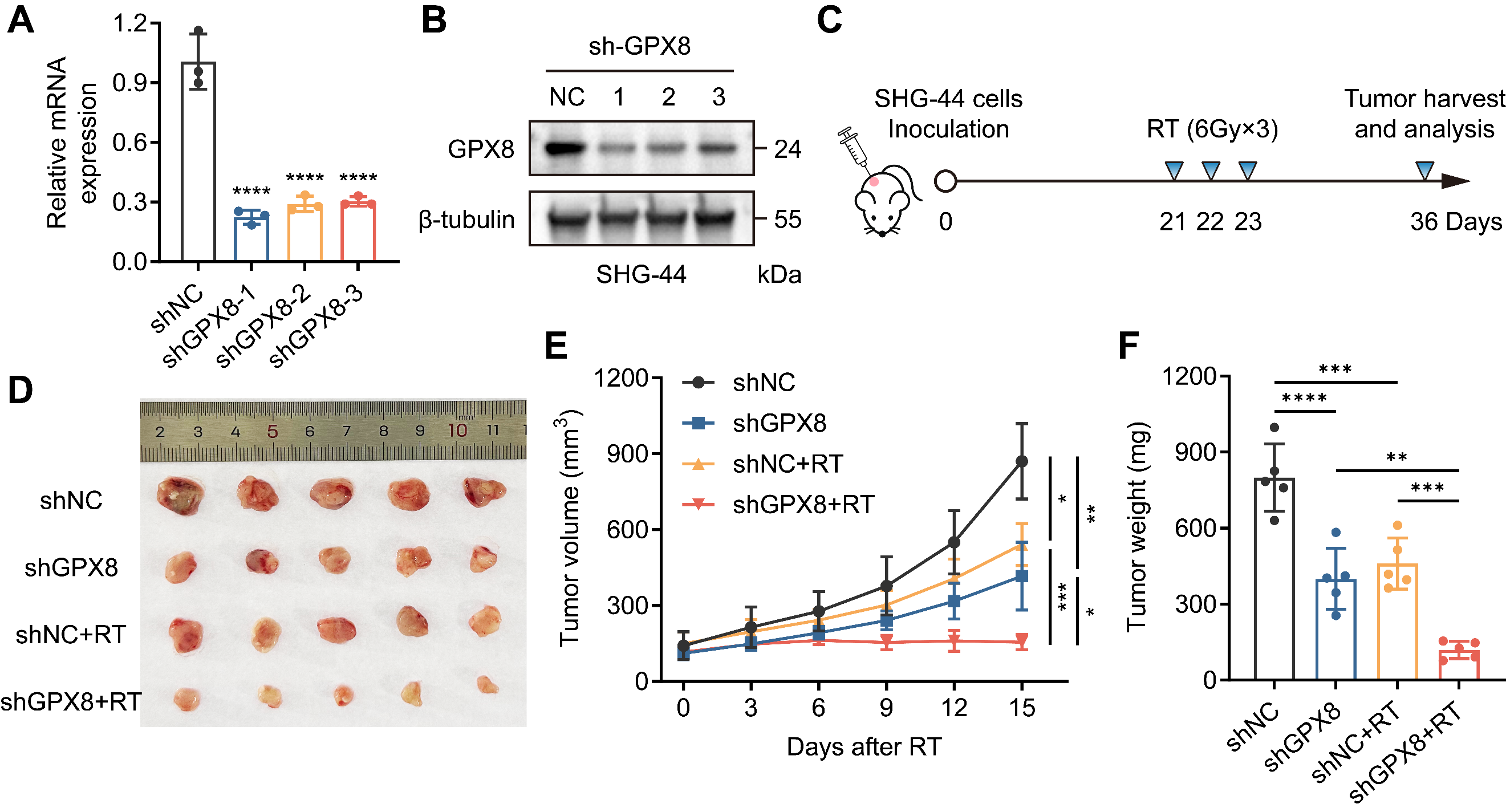


**Table S1. Sequence of primers, siRNAs and shRNAs (5' to 3')**

| **Primer** | β-actin | Forward | ACCCTGAAGTACCCCATCGAG |
| --- | --- | --- | --- |
|  |  | Reverse | AGCACAGCCTGGATAGCAAC |
|  | GPX8 | Forward | CCTCAAGAATGCCAGATGAGTG |
|  |  | Reverse | AGTAAGTGTGTTAATTGACTTTTAGAGTTG |
| **siRNA** | siGPX8-NC | Sense | UUCUCCGAACGUGUCACGUtt |
|  |  | Antisense | ACGUGACACGUUCGGAGAAtt |
|  | siGPX8-1 | Sense | ACUAUAGACAGCAAAACUGCAtt |
|  |  | Antisense | CAGUUUUGCUGUCUAUAGUUCtt |
|  | siGPX8-2 | Sense | AACAGUUCUUCCUUUUGCAUtt |
|  |  | Antisense | GCAAAAGGAAGAACUGUUUCUtt |
|  | siGPX8-3 | Sense | UUGCAAAAGAUUCUACUUCCUtt |
|  |  | Antisense | GAAGUAGAAUCUUUUGCAAGAtt |
| **shRNA** | shGPX8-1 | Forward | gatccGAAGTCATCAGGCCTGACATActcgagTATGTCAGGCCTGATGACTTCtttttt |
|  |  | Reverse | aattaaaaaaGAAGTCATCAGGCCTGACATActcgagTATGTCAGGCCTGATGACTTCg |
|  | shGPX8-2 | Forward | gatccGCCCAAGCAAGGAAGTAGAATctcgagATTCTACTTCCTTGCTTGGGCtttttt |
|  |  | Reverse | aattaaaaaaGCCCAAGCAAGGAAGTAGAATctcgagATTCTACTTCCTTGCTTGGGCg |
|  | shGPX8-3 | Forward | gatccgCCATGAGGGTTTGGTCTCATTctcgagAATGAGACCAAACCCTCATGGtttttt |
|  |  | Reverse | aattaaaaaaCCATGAGGGTTTGGTCTCATTctcgagAATGAGACCAAACCCTCATGGcg |

**Table S2. Antibodies used in this study**

| **Antibody** | **Source** | **Catalog NO.** | **Species Reactivity** |
| --- | --- | --- | --- |
| GPX8 pAb | Proteintech, China | 16846-1-AP | Human, Mouse, Rat |
| β-tubulin pAb | Proteintech, China | 10094-1-AP | Human, Mouse, Rat |
| β-actin mAb | Proteintech, China | 66009-1-Ig | Human, Mouse, Rat |
| GAPDH mAb | Proteintech, China | HRP-60004 | Human, Mouse, Rat |
| E-cadherin pAb | Proteintech, China | 20874-1-AP | Human, Mouse, Rat |
| N-cadherin pAb | Proteintech, China | 22018-1-AP | Human, Mouse, Rat |
| Vimentin pAb | Proteintech, China | 10366-1-AP | Human, Mouse, Rat |
| Snail pAb | Proteintech, China | 13099-1-AP | Human, Mouse, Rat |
| SOX2 pAb | Proteintech, China | 11064-1-AP | Human, Mouse, Rat |
| CD44 pAb | Proteintech, China | 15675-1-AP | Human |
| Caspase-3 pAb | Proteintech, China | 19677-1-AP | Human, Mouse, Rat |
| PARP1 pAb | Proteintech, China | 13371-1-AP | Human, Mouse, Rat |
| Bcl-2 pAb | Proteintech, China | 12789-1-AP | Human |
| Bax pAb | Proteintech, China | 50599-2-Ig | Human, Mouse, Rat |
| γ-H2AX (Ser139) mAb | ABclonal, China | AP0687 | Human, Mouse, Rat |
| RAD51 pAb | Proteintech, China | 14961-1-AP | Human, Mouse |
| Ki-67 pAb | Abcam, UK | ab15580 | Human, Mouse |
| F4/80 pAb | Proteintech, China | 29414-1-AP | Mouse, Rat |
| NKp46 pAb | Abmart, China | TD7599S | Human, Mouse, Rat |
